# *Francisella tularensis* IglG Belongs to a Novel Family of PAAR-Like T6SS Proteins and Harbors a Unique N-terminal Extension Required for Virulence

**DOI:** 10.1371/journal.ppat.1005821

**Published:** 2016-09-07

**Authors:** Mélanie Rigard, Jeanette E. Bröms, Amandine Mosnier, Maggy Hologne, Amandine Martin, Lena Lindgren, Claire Punginelli, Claire Lays, Olivier Walker, Alain Charbit, Philippe Telouk, Wayne Conlan, Laurent Terradot, Anders Sjöstedt, Thomas Henry

**Affiliations:** 1 CIRI, International Center for Infectiology Research, Inserm U1111, CNRS, UMR5308, Lyon, France; 2 University of Lyon, Lyon, France; 3 Ecole Normale Supérieure de Lyon, Lyon, France; 4 Department of Clinical Microbiology, Clinical Bacteriology, and Laboratory for Molecular Infection Medicine Sweden (MIMS), Umeå University, Umeå, Sweden; 5 Institut des Sciences Analytiques, CNRS, UMR 5280, Université de Lyon, Université Claude Bernard Lyon 1, ENS de Lyon, Villeurbanne, France; 6 Université Paris Descartes, Sorbonne Paris Cité, Bâtiment Leriche, Paris, France; 7 Institut Necker-Enfants Malades, Equipe 11: Pathogénie des Infections Systémiques, Paris, France; 8 Laboratoire de Geologie de Lyon; Ecole Normale Supérieure de Lyon, Lyon, France; 9 National Research Council Canada, Human Health Therapeutics Portfolio, Ottawa, Ontario, Canada; 10 Molecular Microbiology and Structural Biochemistry, UMR 5086, CNRS-Université de Lyon, Institut de Biologie et Chimie des Protéines, Lyon, France; University of Illinois, UNITED STATES

## Abstract

The virulence of *Francisella tularensis*, the etiological agent of tularemia, relies on an atypical type VI secretion system (T6SS) encoded by a genomic island termed the *Francisella* Pathogenicity Island (FPI). While the importance of the FPI in *F*. *tularensis* virulence is clearly established, the precise role of most of the FPI-encoded proteins remains to be deciphered. In this study, using highly virulent *F*. *tularensis* strains and the closely related species *F*. *novicida*, IglG was characterized as a protein featuring a unique α-helical N-terminal extension and a domain of unknown function (DUF4280), present in more than 250 bacterial species. Three dimensional modeling of IglG and of the DUF4280 consensus protein sequence indicates that these proteins adopt a PAAR-like fold, suggesting they could cap the T6SS in a similar way as the recently described PAAR proteins. The newly identified PAAR-like motif is characterized by four conserved cysteine residues, also present in IglG, which may bind a metal atom. We demonstrate that IglG binds metal ions and that each individual cysteine is required for T6SS-dependent secretion of IglG and of the Hcp homologue, IglC and for the *F*. *novicida* intracellular life cycle. In contrast, the *Francisella*-specific N-terminal α-helical extension is not required for IglG secretion, but is critical for *F*. *novicida* virulence and for the interaction of IglG with another FPI-encoded protein, IglF. Altogether, our data suggest that IglG is a PAAR-like protein acting as a bi-modal protein that may connect the tip of the *Francisella* T6SS with a putative T6SS effector, IglF.

## Author Summary

*Francisella tularensis* is a highly pathogenic bacterium causing tularemia. Its ability to cause disease is linked to its ability to replicate in the macrophage cytosol. The intracellular life cycle of *Francisella* is controlled by a type VI secretion system (T6SS), which is thought to inject effectors into the host cell to allow bacterial escape into the host cytosol. The molecular mechanisms behind this process are still largely unclear. In this work, we identify IglG as a protein with two important domains, one conserved in proteins from more than 250 bacterial species (DUF4280, renamed here as PAAR-like domain) and one specific for the *Francisella* genus. Using protein sequence analysis and three-dimensional structure predictions, comparative modeling and biochemistry approaches, our data demonstrate that IglG is a metal-binding protein that based on its PAAR-like domain might cap the VgrG spike of the T6SS and act as a membrane-puncturing protein. Furthermore, we identified that the *Francisella*-specific domain is directly involved in forming a protein complex with another virulence protein, IglF. This work, in addition to enhancing the molecular understanding of the *Francisella* T6SS, defines the features of the conserved DUF4280, a novel PAAR-like domain involved in type VI secretion (T6S) of many bacterial species.

## Introduction

*Francisella tularensis* is a Gram-negative bacterium that causes tularemia [1]. The severity of tularemia is highly variable depending on the route of inoculation of the bacterium and the infecting strain. *F*. *tularensis* subspecies *tularensis* is the most virulent subspecies with a 50% lethal dose estimated to be at most 10 bacteria by the intranasal route for humans [2]. A Live Vaccine Strain (LVS), derived from a *F*. *tularensis* subspecies *holarctica* strain, is widely used to study the pathogenesis of tularemia. *F*. *novicida* is another closely related species, which is avirulent for immunocompetent humans but highly virulent in mice. Due to its ability to reproduce the intracellular life cycle of the more virulent subspecies, *F*. *novicida* is widely used as a model system to study tularemia [3].

The ability of *Francisella* strains to cause disease is linked to their ability to replicate within host cells such as macrophages. Upon phagocytosis, *Francisella* escapes from the vacuole to reach the cytosol, where it replicates rapidly. Escape from the vacuole into the host cytosol is dependent on a genomic island termed the *Francisella* Pathogenicity Island (FPI) [4,5]. In addition, the FPI is implicated in the inhibition of macrophage pro-inflammatory response (*e*.*g*. TNF-α secretion) [6,7]. The island is highly conserved between *F*. *tularensis* and *F*. *novicida* with greater than 97% identity at the nucleotide level [8,9], and contains 17 genes, 8 of which encode for proteins that share homology with proteins from type VI secretion systems (T6SS) ([10] and S1 Fig). Two copies of the FPI are present in *F*. *tularensis* strains while a single copy is present in *F*. *novicida* [8]. In the latter species, another genomic island termed the "*Francisella novicida* Island (FNI)" demonstrates some similarities with the FPI suggesting it might encode another atypical T6SS ([10] and S1 Fig).

T6SS are specialized machineries involved in the delivery of toxins and effector proteins to prokaryotic and eukaryotic cells [11–13]. They are functionally related to the bacteriophage contractile tail [14,15]. Contraction of an external sheath consisting of TssB and TssC results in the secretion of an inner tube made of stacks of Hcp protein hexamers. The T6SS is tethered to the bacterial envelope by a membrane complex composed of the inner membrane proteins TssL and TssM/IcmF and the outer membrane lipoprotein TssJ [14]. The Hcp tube is surmounted by a complex made of a VgrG trimer capped with the recently identified PAAR protein [16]. PAAR proteins, originally named TagD [17], are characterized by three proline-alanine-alanine-arginine (PAAR) motifs. A zinc atom bound to three histidines and one cysteine is believed to stabilize their three-dimensional structure [16]. This distal VgrG_3_-PAAR protein complex is thought to act as a membrane-puncturing device allowing the delivery of toxins and effector proteins into the target cell. The mode of secretion may involve physical interactions between the secreted effector and the Hcp, VgrG or PAAR proteins [16,18,19].

Phylogenetic analysis of T6SSs led to the classification of the *F*. *tularensis* T6SS as a unique evolutionary outlier [20]. Indeed, out of the 13 proteins that define the core of prototypical T6SSs, the FPI lacks obvious homologues for at least 5 of them. Furthermore, while the FPI encodes a TssM/IcmF family protein (PdpB), this protein lacks the conserved Walker A box required to bind ATP and to provide energy to the secretion process [9,21]. Although *Francisella* possesses a VgrG protein, it is much smaller than prototypical VgrG proteins [9]. IglC has structural homology with Hcp, the stacking unit of the T6SS inner tube. Yet, it is still unclear whether IglC can also form hexameric rings [22]. Importantly, Clemens *et al*. recently strengthened the T6SS nature of the FPI proteins by demonstrating that IglA and IglB (TssB and TssC homologues, respectively) interact to form the T6SS sheath [23]. While the presence of a functional T6SS encoded by the FPI is now established [9,23,24], it is still largely unknown which FPI genes encode for structural component of the T6SS machinery *per se* and if the FPI proteins demonstrated to be secreted are indeed effector proteins.

IglG is an 18-kDa protein of unknown function encoded by the FPI. Intriguingly, a LVS Δ*iglG* mutant presents delayed kinetics of phagosomal escape compared to parental LVS [7]. Thus, in contrast to *vgrG*, *iglA* (*tssB*), *iglB* (*tssC*) or *iglC* (*hcp*) mutants [25,26], the mutant is not fully confined to the vacuole and replicates efficiently in macrophages [7]. Despite efficient replication, the LVS Δ*iglG* mutant shows delayed cytopathogenicity and impaired inhibition of TNF-α secretion [7,27]. Moreover, IglG is required for virulence of LVS in mice, indicating that this protein is central to the pathogenicity of subsp. *holarctica* [7]. However, it is still unclear whether IglG is a structural component of the T6SS or a T6SS effector. We therefore decided to perform a comparative study using *F*. *novicida* and *F*. *tularensis* subspecies *holarctica* and *tularensis* to identify the nature of IglG. Our results demonstrate that IglG is absolutely required for *F*. *novicida* escape into the host cytosol, triggering of the cytosolic innate immune responses and replication within macrophages. In addition, we demonstrate that IglG is a member of the DUF4280 family, which comprises features of the recently described PAAR protein family located at the tip of the T6SS. While the PAAR motifs are poorly conserved within this family, 4 cysteine residues are highly conserved. These residues were shown to be essential for IglG function in the T6SS and in virulence and to contribute to the coordination of a metal ion. This study thus defines the members of the DUF4280 as novel PAAR-like proteins. In addition, we found that IglG is unique among PAAR-like proteins in bearing an N-terminal extension required for virulence. We demonstrate that IglG, in an N-terminal domain-dependent manner, interacts with the FPI-encoded IglF protein, suggesting that IglG may act as a cargo to connect the tip of the *Francisella* T6SS with other T6SS proteins/effectors. Finally, the importance of IglG for the virulence of *F*. *novicida* as well as the highly virulent *F*. *tularensis* SCHU S4 strain was demonstrated *in vivo*.

## Results

### IglG and proteins from the DUF4280 family feature a predicted PAAR-like structure

**Fig 1:**
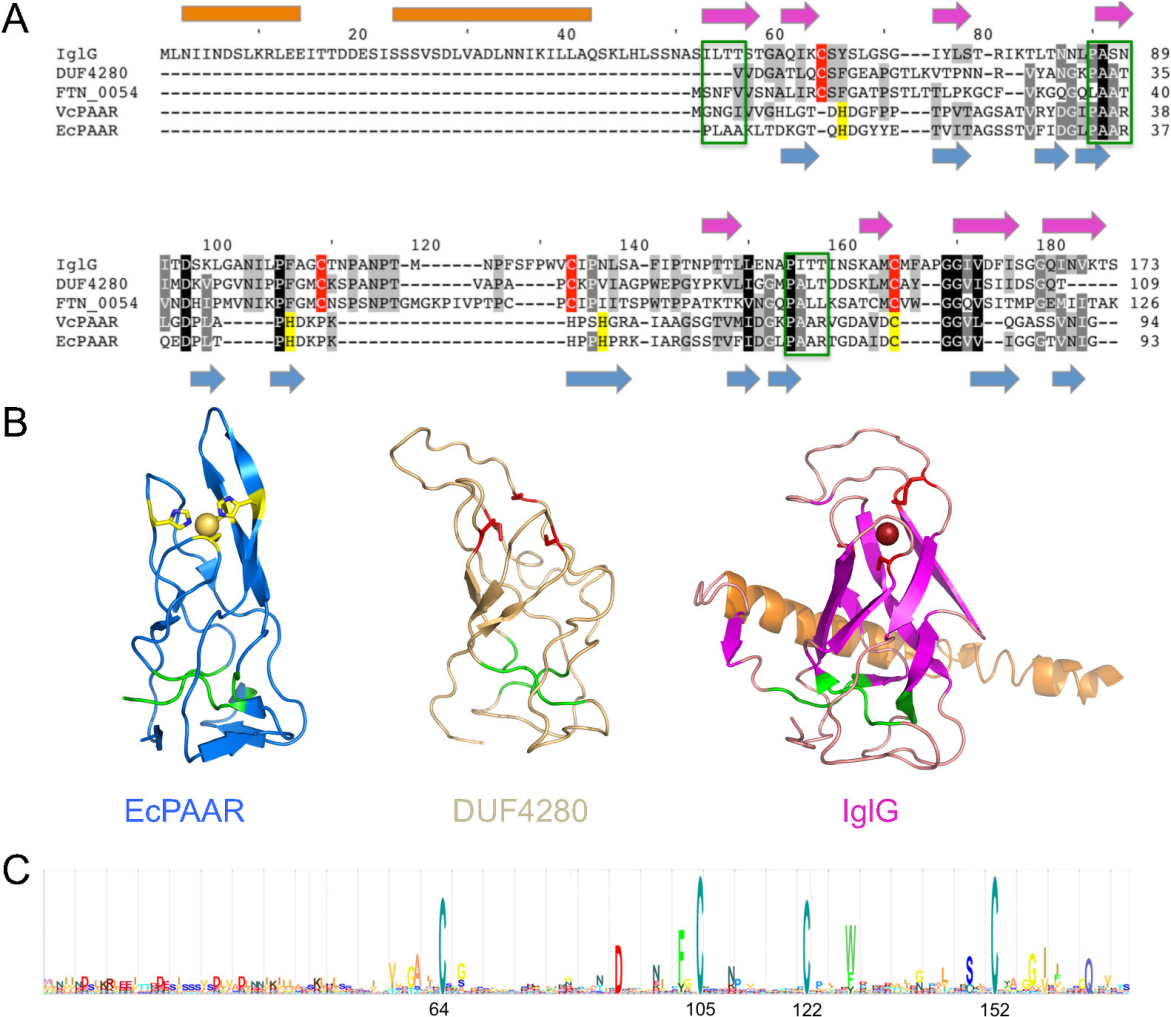
IglG, a DUF4280-containing protein, is a structural homologue of PAAR proteins with four conserved cysteines potentially implicated in metal ion binding. (A) Protein sequence alignment of IglG, DUF4280 (consensus sequence), FTN_0054, VcPAAR (pdb code 4JIV) and EcPAAR (4JIW). Predicted secondary structure elements of IglG are indicated above the sequence (orange rectangles for α-helices and magenta arrows for β-strands). Secondary structures of EcPAAR are indicated below the alignment in blue. Green boxes indicate the PAAR motifs, residues binding Zn in VcPAAR and EcPAAR are shaded in yellow. Conserved cysteines in the sequences of IglG, FTN_0054 and DUF4280 are shaded in red. (B) A comparison of the EcPAAR structure (blue) with homology models of DUF4280 (wheat) and IglG (magenta). Segments colored in green in the three structures correspond to the PAAR motifs. Spheres indicate the position of Zn ion (colored in yellow) in EcPAAR and the putative metal binding site in IglG. The side chains of metal binding residues are shown as ball and stick and colored in yellow (EcPAAR) or red (DUF4280 and IglG). Structures are otherwise colored as in A. (C) Profile hidden Markov model (HMM) generated by iterative searches with IglG sequence using the Jackhmmer software. The positions of the four conserved cysteine residues within IglG are indicated.

To gain insight into the function of IglG, we performed a bioinformatic analysis. A BLAST search using the IglG sequence identified a conserved domain of unknown function (DUF4280; IPR025460; PF14107) in the C-terminus of the protein (residues 59–173) (Fig 1A). DUF4280 is a domain present in numerous proteins of unknown function and is found in more than 250 different bacterial species. One of the DUF4280 proteins, Fjoh_3275/Fte1 from *Flavobacterium johnsoniae* was recently predicted to adopt a fold closely related to the PAAR domain [28] suggesting that other proteins containing the DUF4280 domain might also share these sequence signatures. We thus performed homology modeling using the Phyre [29] and I-Tasser [30] servers with IglG or the DUF4280 consensus sequence as templates. Both servers clearly identified the structures of *Escherichia coli* or *Vibrio cholerae* PAAR proteins as suitable templates for modeling and produced high confidence models (e.g. 85% confidence according to Phyre) for the DUF4280 consensus sequence and for IglG residues 59–173 (Fig 1B). The models generated by I-Tasser for residues 59–173 of IglG and for the DUF4280 consensus sequence were all very similar to that of a conical β-barrel fold (Fig 1B). The three PAAR motifs were, however, not strictly conserved neither in IglG, nor in the DUF4280 proteins (Fig 1A). Yet, one to one threading [29] with a typical PAAR protein from *V*. *cholerae* (VCA0105, here denoted VcPAAR) identified partial sequence conservation for two out of three of these motifs (Fig 1A). Our confidence in the model was strengthened by a molecular dynamics simulation with a 400 ns trajectory on the IglG C-terminus structure presented above. Indeed, the root mean square fluctuation obtained (RMSF = 0.27 ^+^/_-_ 0.11) was very similar to the RMSF of a well-characterized structural domain (RMSF_SH3 domain_ = 0.24 ^+^/_-_ 0.06) (S2 Fig and S1 Movie). This simulation thus suggests that the predicted protein fold would be stable over time.

We were unable to purify IglG or any of the other DUF4280 proteins tested (FTN_0054 from *F*. *novicida*, PA_2375 from *P*. *aeruginosa* and ROD_34101 from *Citrobacter rodentium*) in soluble form and in sufficient amount to perform crystallization assays. This observation is in line with the described low solubility of PAAR proteins [16] and highlights the challenge of working on individual T6SS proteins out of the context of the whole machinery. However, His_6_-tagged IglG could be purified from in *E*. *coli* under denaturing conditions and refolded using step-wise dialysis. Importantly, the analysis of the refolded IglG protein by circular dichroism (CD) spectroscopy supported our *in silico* analysis since the proportion of α-helices and β-strands determined with this method (25% and 22%, respectively) was highly similar to the one predicted in our model (23% and 22%, respectively). Altogether, these data demonstrated that IglG and the members of the DUF4280 family define a novel family of PAAR-like proteins formed by a β-barrel.

### IglG is essential for *F*. *novicida* phagosomal escape and replication within macrophages

**Fig 2:**
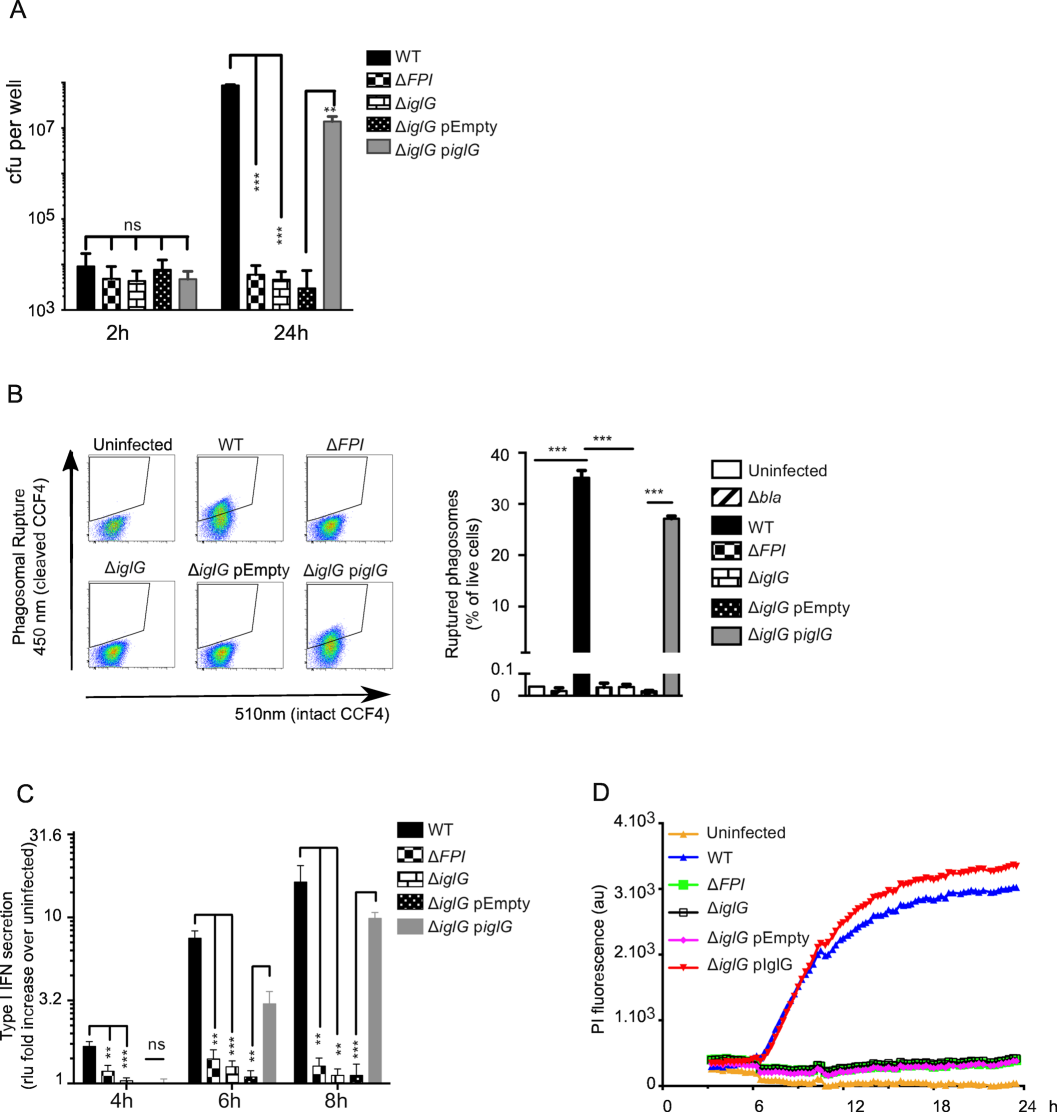
IglG is required for intracellular replication, phagosomal escape and triggering of cytosolic innate immune responses. (A) J774 macrophages were infected at an MOI of 1 with the indicated *F*. *novicida* strains and the intracellular burden was assessed by determination of viable counts at 2 and 24 h. (B) Phagosomal rupture in BMDMs infected with the indicated strains at an MOI of 100 was determined at 2 h by flow cytometry using the β-lactamase/CCF4 assay. Concatenates from three samples are shown for each strain after exclusion of doublets and dead cells. Quantification is shown in the right panel using the gating strategy presented in the left panel. (C) Type I IFN secretion in the culture supernatant of BMDMs infected with the indicated strains at an MOI of 1 was determined by the ISRE-luciferase bioassay and normalized to the value of the bioassay in uninfected macrophages. (D) Cell death of BMDMs infected with the indicated strains at an MOI of 1 was monitored in real time in the presence of propidium iodide by fluorescence readings every 15 min. (A-D) Mean and (A-C) SD of triplicates from one experiment representative of 3 independent experiments are shown. Unpaired *t*-tests were performed, two-tailed *P*-values are shown (ns, not significant; **, *P* ≤ 0.01; ***, *P* ≤ 0.001).

To investigate the role of IglG for the T6SS, we made a full-length deletion of *iglG* (FTN_1314) in *F*. *novicida* strain U112. The *F*. *novicida* Δ*iglG* mutant was unable to replicate in J774 macrophages (Fig 2A) as well as in primary bone marrow-derived macrophages. This phenotype resembled that of a mutant lacking the whole pathogenicity island (Δ*FPI*) [31] and could be complemented by expression of the IglG protein *in trans* (Fig 2A). The main function of the FPI during the *Francisella* intracellular life cycle is to promote the escape of *Francisella* into the host cytosol [32]. We thus investigated the occurrence of phagosomal rupture by using the β-lactamase/CCF4 assay [33,34]. IglG was required for vacuolar escape since we did not observe any phagosomal rupture in Δ*iglG* mutant-infected macrophages at 2 h post-infection (Fig 2B). At this time point, phagosomal rupture could be detected in more than 25% of the cells infected with the WT strain or the complemented Δ*iglG* mutant strain. As expected, no phagosomal rupture was detected in cells infected by the Δ*FPI* mutant or by a Δ*bla* mutant lacking a functional *F*. *novicida* β-lactamase [35]. In addition, we did not observe any phagosomal rupture in macrophages infected with the Δ*iglG* mutant strain even at 18 h (S3 Fig) suggesting that in *F*. *novicida*, IglG is essential for the escape from the vacuole. Escape of *F*. *novicida* into the host cytosol triggers cytosolic innate immune responses, namely secretion of type I IFN [36] and activation of the AIM2 inflammasome [37]. In agreement with the lack of phagosomal escape, the *F*. *novicida* Δ*iglG* mutant failed to induce type I IFN secretion (Fig 2C) and inflammasome-mediated death as measured by real-time propidium iodide incorporation (Fig 2D). Similarly, the Δ*iglG* mutant was not cytopathogenic towards J774 macrophages even at 48 h as determined by measuring the release of LDH into the culture supernatant (S4 Fig). Altogether, these data demonstrate that in *F*. *novicida*, deletion of *iglG* leads to the same phenotype as the deletion of *iglC* (the *hcp* homologue) or of *vgrG*, in agreement with a PAAR-like role of IglG in the T6S machinery.

### IglG is secreted in a T6SS-dependent manner and is required for secretion of the Hcp homologue, IglC

**Fig 3:**
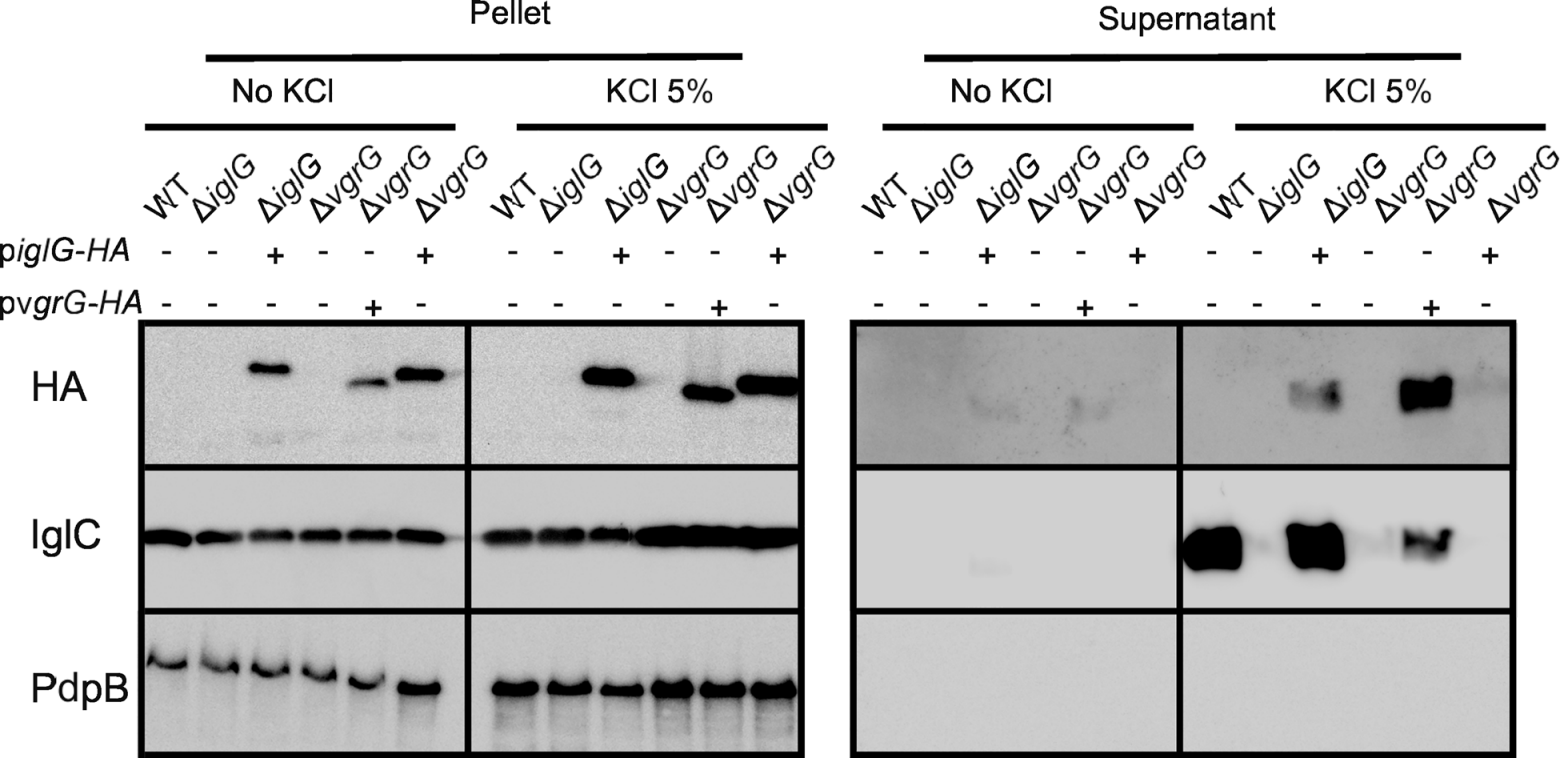
IglG is secreted in a T6SS-dependent manner and is required for secretion of the Hcp homologue, IglC. The amount of IglC and HA-tagged IglG or VgrG proteins was assessed in the concentrated supernatant fractions or in bacterial pellets of the indicated strains grown in the presence or absence of 5% KCl. The non-secreted inner membrane protein PdpB was included as a control. One experiment representative of 2 independent experiments is shown.

Based on its PAAR-like nature, we expected IglG to be secreted as a key component of the Hcp/IglC tube. We first attempted to monitor IglG secretion into host cells by flow cytometry using the β-lactamase reporter [24]. Using this method and in agreement with our previous microscopy-based assay [24], we could not detect translocation of IglG, VgrG, IglI or IglF, although PdpE and IglC were readily secreted (S5 Fig, left panel). Since all fusion proteins were expressed (S5 Fig, right panel), this suggests that the large tag may have an adverse effect on the secretion of some FPI proteins in *F*. *novicida*. We thus switched to a recently described *in vitro* secretion assay in which addition of KCl triggers T6SS-dependent secretion [23]. Upon KCl addition, IglG was secreted into the culture supernatant (Fig 3). As expected VgrG was also specifically secreted during this condition [23], while PdpB, the inner membrane-associated TssM homologue, was not detected in the supernatant (Fig 3). Importantly, IglG secretion was abolished in a Δ*vgrG* mutant indicating that a functional T6SS is required for IglG export. Similarly, we could not detect secretion of IglG or VgrG in the absence of KCl indicating that environmental cues associated with T6SS activation are required for their secretion.

Deletion of PAAR proteins highly reduces Hcp secretion by *V*. *cholerae* and *Acinetobacter baylyi* [16]. We thus tested whether a deletion of *iglG* in *F*. *novicida* would impair secretion of the Hcp homologue, IglC. Indeed, secretion of IglC was absent in the Δ*iglG* mutant, although in a few experiments low levels of IglC were still detected in the mutant (Fig 3 and S6 Fig). Similarly, IglC was not detected in the supernatant of the Δ*vgrG* mutant or in the supernatant of the WT strain if KCl was absent from the growth medium (Fig 3). Importantly, expression of IglG and VgrG *in trans* restored IglC secretion by Δ*iglG* and Δ*vgrG* mutants, respectively (Fig 3). Altogether, these data, together with the results from the bioinformatic analysis, are consistent with a PAAR-like role of IglG in the *F*. *novicida* T6SS.

### The four cysteine residues conserved in DUF4280 proteins are required for the function of IglG in *F*. *novicida* and contribute to metal binding

**Fig 4:**
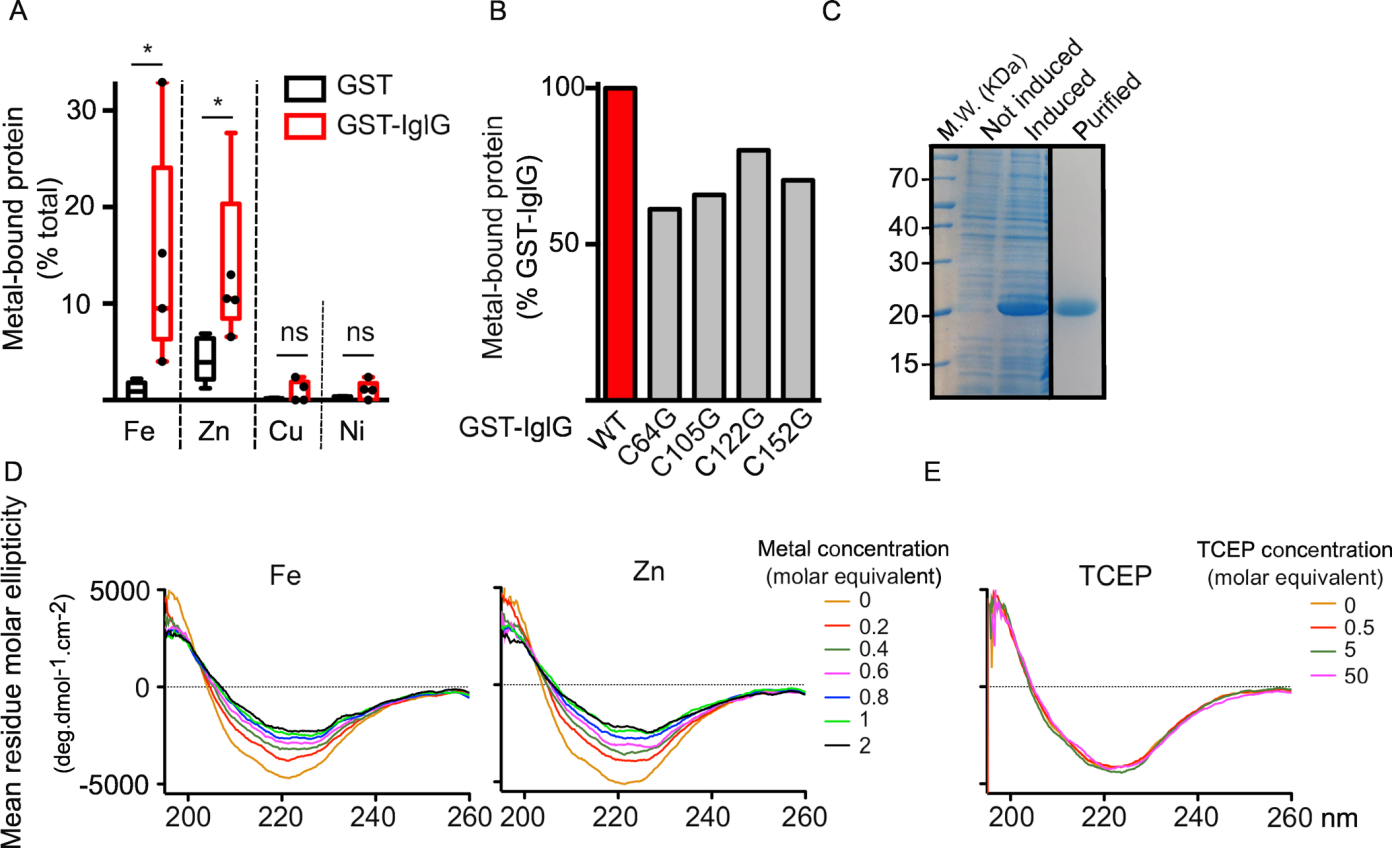
IglG is a metal-binding PAAR-like protein. (A) Metal content of purified GST-IglG or GST proteins was determined by ICP-MS. Values for Iron (Fe), Zinc (Zn), Copper (Cu) and Nickel (Ni) are shown. Box and whiskers (Min to Max) are shown with each point representing the metal to protein ratio for independent protein expression and purification processes (n = 5). (B) Metal content of purified GST-IglG or the indicated GST-IglG cysteine mutant proteins was determined by ICP-MS. The metal content (iron plus zinc) of each GST-IglG cysteine mutant was normalized to the metal content of the GST-IglG protein expressed and purified at the same time. (C) Purified His_6_-IglG protein is shown by coomassie blue staining (lane 3) after purification under denaturing conditions and refolding by step-wise dialysis. Whole cell lysates before (lane 1) and after IPTG induction (lane 2) are shown. (D, E) The conformation of His_6_-IglG protein refolded in the presence of EDTA was analyzed by circular dichroism spectroscopy upon addition of increasing concentrations of FeSO_4_ and ZnSO_4_ (D), or increasing concentrations of TCEP (E) to reduce the potential disulfide bonds (concentrations are expressed as molar equivalent).

PAAR proteins bind Zn^2+^ via 3 histidine and 1 cysteine residues (Fig 1A and 1B). The central Zn^2+^ ion is thought to be important to stabilize the protein structure [16]. While the cysteine residue is conserved in IglG (Cys 152) and in the PAAR-like proteins, no histidines are present neither in IglG nor in the DUF4280 consensus sequence (Fig 1A). However, both the sequence alignment and the 3D models suggest that this residue, together with 3 other cysteines (Cys 64, 105, 122 in IglG) could potentially form the metal-binding site of the PAAR-like proteins (Fig 1A and 1B). The strong conservation of these residues in IglG homologues was highlighted by iterative blast searches using the Jackhmmer software (Fig 1C), suggesting that their presence is essential to the function of these PAAR-like proteins. To determine if IglG is able to bind metal ions, we expressed IglG fused to GST and tested whether the purified fusion protein could bind metal by using inductively coupled plasma mass spectrometry (ICP-MS). Although we did observe some variability in metal binding between the different batches of purified proteins (five independent experiments are shown), iron and zinc were specifically associated with GST-IglG. In contrast, we detected only trace amounts of nickel and copper while the other metals tested (e.g. Mn, Co) were undetectable (Fig 4A). The observed ratio between iron and zinc binding to IglG ([Fe]/[Zn] = 1.6) suggests that, under our conditions (see discussion), iron binding to GST-IglG is slightly favored over zinc binding. Of note, the same experiments performed on a GST-fusion of a typical PAAR protein from *Pseudomonas aeruginosa* (PA_0824) demonstrated a similar proportion of molecules binding metal (about 30% of the total purified molecules) but with a strong binding preference for zinc ([Fe]/[Zn] = 0.3) (S7 Fig). Experiments performed in parallel with the GST control protein identified only traces of iron and low concentrations of zinc (equivalent in molarity to 4.2% of the total number of GST molecules).

The contribution of each IglG cysteine in Fe/Zn binding was evaluated using purified mutant proteins and ICP-MS. Although we could not directly compare the metal-binding capacity of mutant proteins purified on different days, the direct comparison of each mutant IglG protein to the WT IglG protein purified the same day demonstrated a 20 to 40% reduction in metal binding (Fig 4B). These results suggest that the four cysteine residues contribute to the ability of IglG to bind metal ion but that mutating a single cysteine residue is not sufficient to abolish metal binding *in vitro*.

To strengthen our findings, we performed a metal binding assay using circular dichroism (CD) spectroscopy [38]. CD spectra of IglG protein purified under denaturing conditions and refolded as described above (Fig 4C) were recorded upon addition of different concentrations of Fe^2+^. We observed a modification of the CD spectrum indicative of a change of conformation upon addition of 0.2 molar equivalent of iron (i.e. 0.2 molecule of iron per molecule of IglG) (Fig 4D). The CD spectra recorded at increasing iron concentrations intersected at a single point (isodichroic point) indicating that addition of increasing concentrations of iron led to a change in the proportion of two unique conformations. Importantly, no further modifications of the CD spectra were observed after addition of one equivalent of iron strongly suggesting that the modifications observed resulted from the binding of one single iron ion per protein. Similar observations were obtained with Zn^2+^ (Fig 4D), Mg^2+^ and Co^2+^ (S8 Fig) suggesting, as observed in the ICP-MS experiments presented above, a promiscuous ability of IglG to bind divalent cations. No change of conformation was observed upon addition of up to 50 molar equivalent of TCEP (Tris (2-carboxyethyl) phosphine), a reducing agent suitable for CD application (Fig 4E), which suggests that the cysteine residues are not involved in forming disulfide bonds within the purified IglG protein.

Altogether, these experiments demonstrate that IglG binds divalent cations. While this remains to be confirmed with other PAAR-like proteins, it suggests that PAAR and PAAR-like proteins have a conserved ability to bind a metal ion to stabilize this specific protein structure. The nature of the bound metal ion remains to be determined *in vivo*.

**Fig 5:**
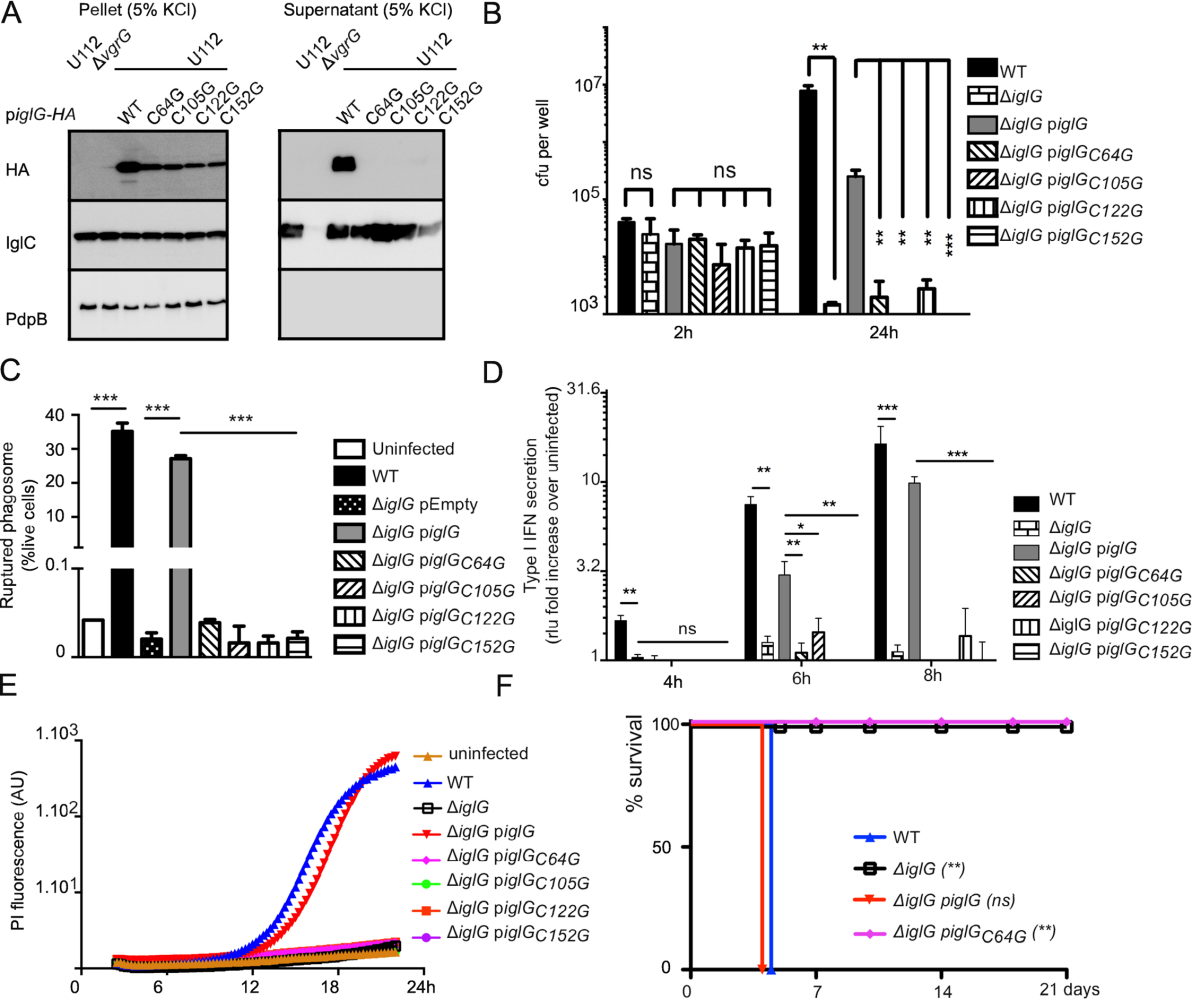
The four cysteines conserved in PAAR-like proteins are required for IglG secretion and function. (A) Levels of 2HA-tagged IglG variants were assessed in the concentrated supernatant fractions or in bacterial pellets of the indicated strains grown in the presence of 5% KCl. Since all of the IglG constructs were expressed in the U112 background, IglC was similarly secreted in these strains. The non-secreted inner membrane protein PdpB was included as a lysis control. (B) J774 macrophages were infected at an MOI of one with the indicated strains. Intracellular burden was assessed by determination of viable counts at 2 and 24 h. (C) Phagosomal rupture in BMDMs infected with the indicated strains at an MOI of 100 was determined at 2 h by flow cytometry using the β-lactamase/CCF4 assay. (D) Type I IFN secretion in the supernatant of BMDMs infected with the indicated strains at an MOI of 1 was determined by the ISRE-luciferase bioassay and normalized to the value of the bioassay in uninfected macrophages. (E) Cell death of BMDMs infected with the indicated strains at an MOI of 1 was monitored in real time in the presence of propidium iodide by fluorescence readings every 15 min. (F) Survival of mice following intradermal challenge with U112 strains. Mice were challenged with the dose indicated in Materials and Methods and monitored for 21 days for signs of illness. (A-E) Mean and (A-D) SD of triplicates from one experiment representative of 2 to 3 independent experiments are shown. Unpaired *t*-tests were performed; two-tailed *P*-values are shown. (F) Mantel-Cox test was performed, *P*-values for the comparison with WT survival curves are shown (ns: not significant; *, *P* ≤ 0.05; **, *P* ≤ 0.01; ***, *P* ≤ 0.001).

To next investigate the role of the cysteines for PAAR-like protein function in the T6SS, we mutated each of these residues in IglG. We first assessed whether the conserved cysteine residues were required for IglG and IglC secretion using the KCl secretion assay. While the wild-type IglG was easily detected in the supernatant in the presence of KCl, we did not detect secretion of any of the four cysteine mutant proteins when expressed in U112 (Fig 5A).

Similarly, when each of the mutant proteins was expressed in the Δ*iglG* mutant, the secretion of the Hcp homologue IglC was strongly decreased and at the same level as in the non-complemented Δ*iglG* mutant (S6 Fig). Moreover, mutating each individual cysteine completely abolished the ability of the Δ*iglG* mutant to replicate in macrophages (Fig 5B), to escape from the phagosome (Fig 5C), and to trigger cytosolic innate immune responses (Fig 5D and 5E). To further demonstrate the key roles of the conserved cysteines, we selected the first cysteine mutant (Cys 64) to assess its virulence in mice. Whereas intradermal injection of U112 or of the Δ*iglG* mutant expressing full-length IglG-2HA killed mice within 4 days, all mice given the Δ*iglG* mutant, or the Δ*iglG* mutant complemented with IglG_C64G_-2HA survived until the end of the experiment (Fig 5F). Altogether, these findings highlight the importance of the four conserved cysteine residues to maintain the T6SS-dependent secretion of IglG and of the Hcp homologue IglC as well as the functional role of IglG in *F*. *novicida* virulence. Our data suggest that the role of the conserved cysteines in the PAAR-like proteins may be linked to their metal-binding ability, which is likely to stabilize the PAAR-like fold.

### IglG is a distinct member of the PAAR-like family by featuring a unique N-terminal alpha helix extension associated with *F*. *novicida* virulence

**Fig 6:**
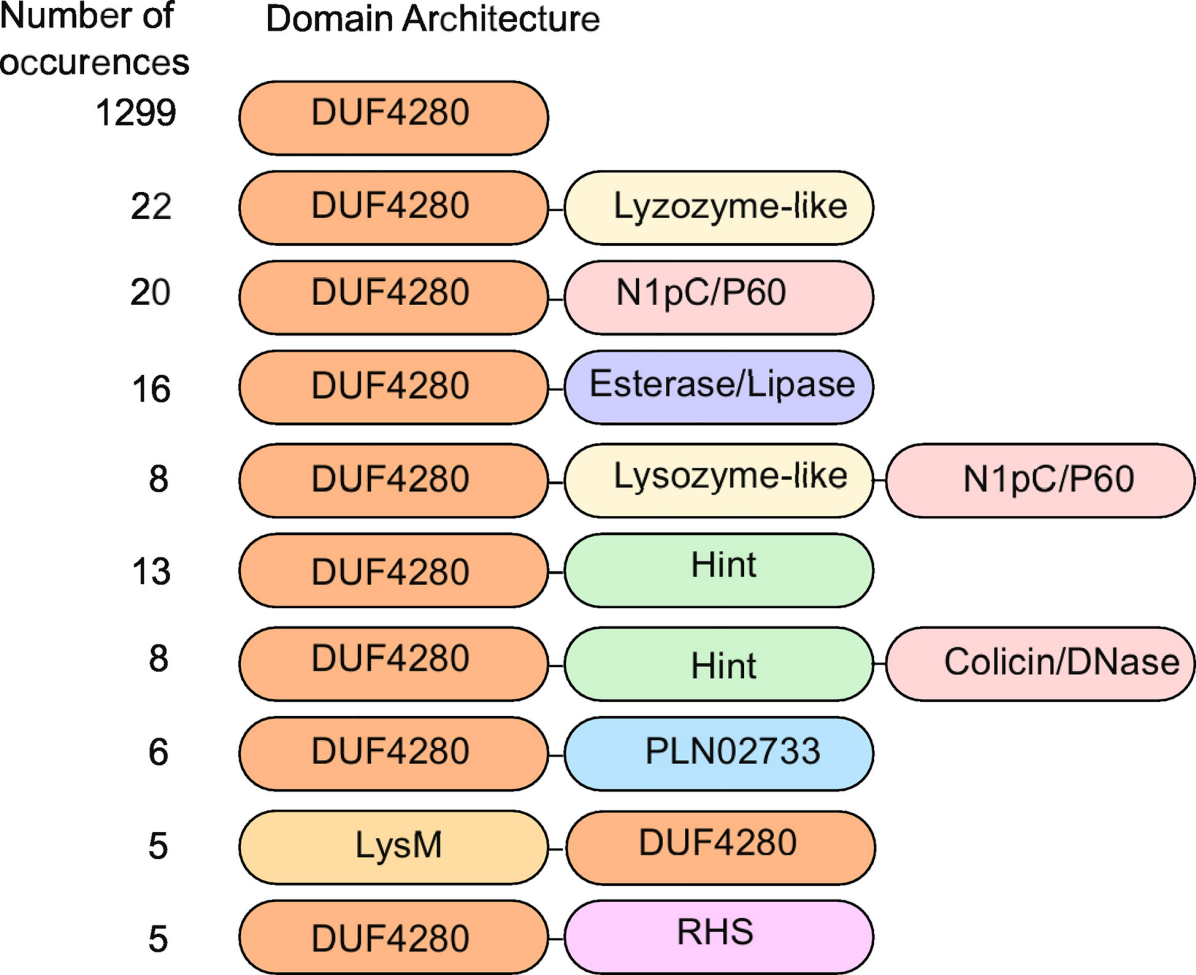
A subset of PAAR-like proteins is extended by T6SS effector domains. The 10 most prevalent domain architectures of DUF4280-containing proteins are shown. Data were retrieved from CDART using the IglG protein sequence as input. The name of the DUF4280-associated domain (and when required a short description) is given below together with the superfamily accession number. Lysozyme-like superfamily (cl00222); NLpC_P60 (cl21534), a family of cell-wall peptidases [40]; Esterase-lipase superfamily (cl21494); Hint superfamily (cl22434): Hedgehog/Intein domain; Colicin-DNase superfamily (cl15861); PLN02733 superfamily (cl22774): phosphatidylcholine-sterol O-acyltransferase; LysM superfamily (cl00107). RHS repeats (cl11982 & cl14012) [39].

Mirroring PAAR proteins [16], a subset of PAAR-like proteins harbors N- or C-terminal extensions including rearrangement hotspot (RHS) repeats [39], toxin and hydrolase domains (Fig 6); suggesting they could act as T6SS effectors.

**Fig 7:**
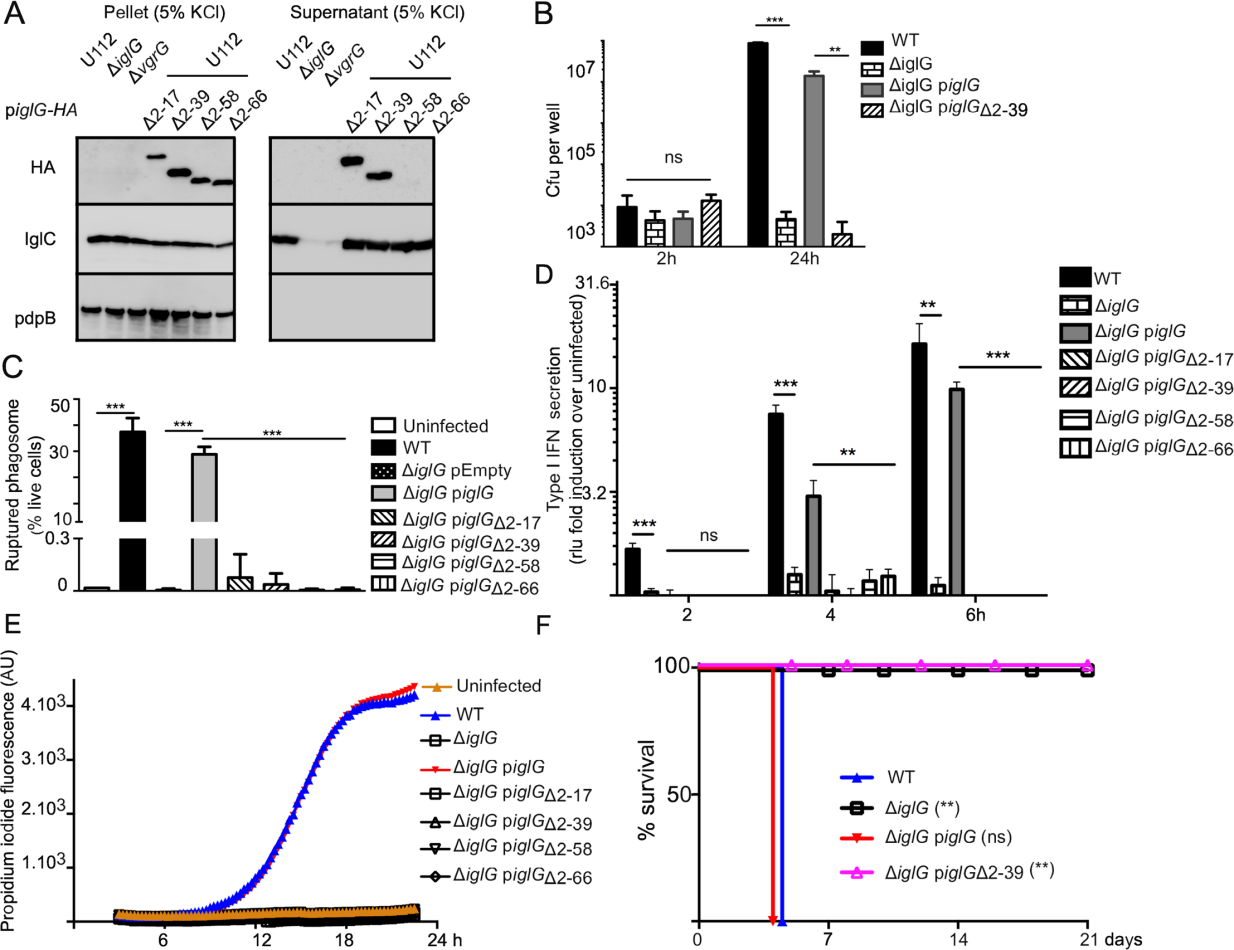
The first 39 residues of the N-terminal extension of IglG are dispensable for IglG secretion but required for IglG function in virulence. (A) Levels of IglC or the indicated HA-tagged proteins were assessed in the concentrated supernatant fractions or in bacterial pellets of the indicated strains grown in the presence of 5% KCl. Since all of the IglG constructs were expressed in U112 background, IglC is similarly secreted in these strains. The non-secreted inner membrane protein PdpB was included as a control for cell-lysis. (B) J774 macrophages were infected at an MOI of one with the indicated strains. Intracellular burden was assessed by determination of viable counts at 2 and 24 h. (C) Phagosomal rupture in BMDMs infected with the indicated strains at an MOI of 100 was determined at 2 h by flow cytometry using the β-lactamase/CCF4 assay. (D) Type I IFN secretion in the supernatant of BMDMs infected with the indicated strains at an MOI of 1 was determined at the indicated time points by the ISRE-luciferase bioassay and normalized to the value of the bioassay in uninfected macrophages. (E) Cell death of BMDMs infected with the indicated strains at an MOI of 1 was monitored in real time in the presence of propidium iodide by fluorescence readings every 15 min. (F) Survival of mice following intradermal challenge with U112 derivatives. Mice were challenged with the dose indicated in Materials and Methods and monitored for 21 days for signs of illness. (B-E) Mean and (B-D) SD of triplicates from one experiment representative of 2 to 3 independent experiments are shown. Unpaired *t*-tests were performed; two-tailed *P*-values are shown. (F) Mantel-Cox test was performed, *P*-values for the comparison with WT survival curves are shown (ns: not significant; **, *P* ≤ 0.01; ***, *P* ≤ 0.001).

Interestingly, the primary sequence alignment (Fig 1A) and the three-dimensional structure modeling (Fig 1B) both clearly identified an extension of the PAAR-like domain at the N-terminus of IglG. This extension, encompassing residues 1 to 57, is not present in the other PAAR-like protein (FTN_0054) encoded in the FNI (Fig 1A and S1 Fig). The specificity of the N-terminal extension is strengthened by the lack of homology to other PAAR-like proteins (Fig 1A) or to any polypeptides outside of the genus *Francisella* (except for an IglG-like protein in *Piscirickettsia salmonis*, a species closely related to *Francisella* [41], S9 Fig). The N-terminal extension of IglG is predicted to contain predominantly α-helices (Fig 1A and 1B). The tertiary structure of this domain was modeled with poor reliability, resulting in one or two helices clearly protruding outside the core PAAR domain albeit with various orientations in the different models (S10 Fig). To experimentally assess the role of this extension in T6SS function and virulence, we engineered a series of N-terminal deletion mutants deleting one or both of the two predicted α-helices (IglG_Δ2–17_ and IglG_Δ2–39_, respectively), the whole N-terminal extension (IglG_Δ2–58_) or the whole extension together with the first 8 residues of the PAAR-like domain (IglG_Δ2–66_). We first analyzed secretion of the IglG proteins *in vitro* in the presence of 5% KCl, using the strain U112 to overcome any problem with lack of complementation (Fig 7A). Interestingly, the two α-helices of the N-terminal extension were not required for *in vitro* secretion, since IglG_Δ2–17_ and IglG_Δ2–39_ were both efficiently secreted. In contrast, longer deletions (Δ2–58 or Δ2–66) abolished IglG secretion. When tested in the macrophage assays, even the smallest deletion (Δ2–17) failed to complement the Δ*iglG* mutant for intracellular replication (Fig 7B), escape from the vacuole (Fig 7C), or triggering of the cytosolic innate immune responses (Fig 7D and 7E). Finally, the importance of the N-terminal extension was validated *in vivo*. Indeed, the Δ*iglG* mutant expressing IglG_Δ2-39_-2HA was unable to kill mice, while, as previously mentioned, the Δ*iglG* mutant expressing IglG-2HA killed 100% of the infected mice within 4 days (Fig 7F). Importantly, these results dissociate for the first time secretion of a FPI protein from its requirement for *F*. *novicida* virulence. Furthermore, these results highlight two distinct domains of IglG critical for protein function; a unique α-helical N-terminal extension dispensable for secretion but required for virulence and a conserved PAAR-like C-terminal domain, which is a key component of the T6SS.

### The four conserved cysteine residues and the N-terminal α-helices are required for IglG function in the *F. tularensis* LVS and *F. tularensis* SCHU S4 strains

**Fig 8:**
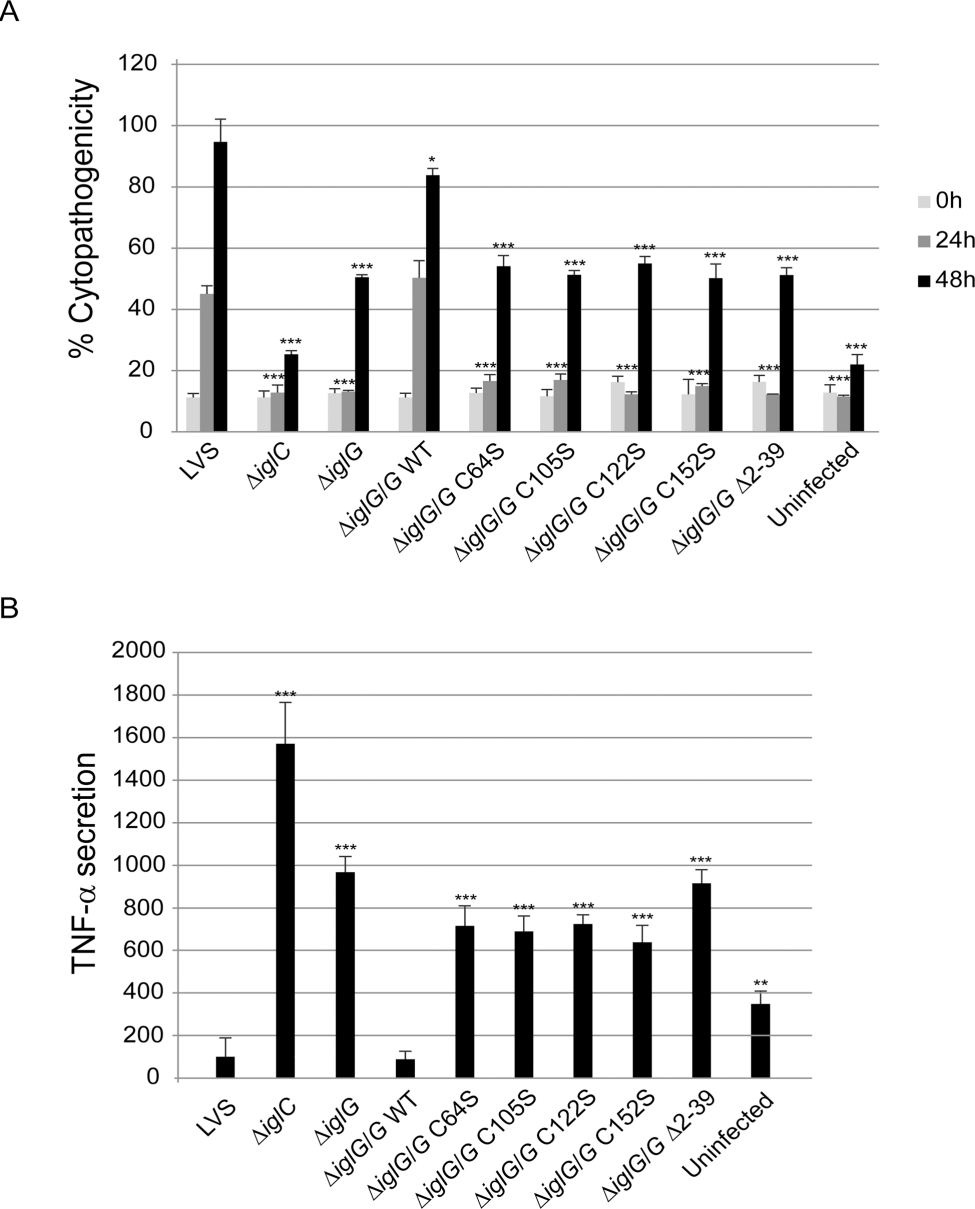
The conserved cysteines and the first 39 residues of the N-terminal extension of IglG are required for IglG function in LVS. LDH release (A) and TNF-α secretion (B) from *F*. *tularensis* LVS-infected J774 cells. (A) Culture supernatants of infected J774 cells were assayed for LDH activity at 0, 24 and 48 h and the activity was expressed as a percentage of the level of non-infected lysed cells (positive lysis control). Values of triplicate wells (means +/- SD) from one representative experiment of two are shown. The asterisks indicate that the cytopathogenicity levels were different from those of LVS-infected cells at a given time point as determined by a 2-sided *t*-test with equal variance (*, *P* ≤ 0.05; ***, *P* ≤ 0.001). (B) J774 cells left uninfected or infected with *F*. *tularensis* at an MOI of 500 for 2 h, were washed and subsequently incubated in the presence of *E*. *coli*-derived LPS (50 ng/ml) for an additional 2 h. The average TNF-α secretion (%) compared to LVS, which was set as 100%, and the SD of quadruple samples (n = 4) from one representative experiment out of two is shown. The asterisks indicate that the cytokine levels were significantly different than those of LVS-infected cells as determined by a 2-sided *t*-test with equal variance (**, *P* ≤ 0.001; ***, *P* ≤ 0.001).

The IglG proteins are highly conserved between *Francisella* species with greater than 98% identity (S11 Fig). Therefore, it was puzzling that the phenotypes of *F*. *novicida* and LVS Δ*iglG* mutants (the latter being deleted of the two *iglG* copies [7]) differed so strongly with regard to phagosomal escape, replication and cytopathogenicity. Indeed, the initial defect in phagosomal escape observed for the LVS Δ*iglG* mutant is only transient, eventually leading to efficient intracellular replication but a delayed cytopathogenic response [9,25]. We thus decided to check whether the critical features of the *F*. *novicida* IglG protein were also required for IglG function in LVS and in the highly virulent *F*. *tularensis* SCHU S4 strain. We first analyzed whether *F*. *novicida*-derived IglG could restore the defects of LVS Δ*iglG* with regard to cytopathogenicity and inhibition of LPS-induced TNF-α secretion [9]. While the wild-type *F*. *novicida* protein was equally competent as LVS-derived IglG at restoring the defects, *F*. *novicida* derived mutant proteins IglG_Δ2–39_, IglG_C64G_, IglG_C105G_, IglG_C122G_ and IglG_C152G_ were all unable to restore the defects of LVS Δ*iglG*. We also confirmed these findings by constructing an IglG_Δ2–39_ mutation within the LVS-derived IglG protein, and by individually exchanging the cysteines of LVS-derived IglG to either serine or alanine. Again, only the wild-type IglG construct was able to restore the defects of the LVS Δ*iglG* mutant back to LVS levels, while the phenotype of Δ*iglG* expressing either of the mutant variants was very similar to that of the non-complemented Δ*iglG* mutant (Fig 8A and 8B). This was not a consequence of a loss of expression, since all of the mutant forms were efficiently expressed (S12 Fig). We were unable to induce type VI secretion *in vitro* for LVS or SCHU S4 using high KCl concentrations since the two strains were severely impaired for growth in such a medium. As previously described by a microscopy-based assay [24], *in cellulo* secretion of IglC-TEM was clearly detected by flow cytometry when expressed in a LVS Δ*iglG* mutant (S13 Fig). Similarly, VgrG-TEM was secreted by the LVSΔ*iglG* mutant at a significant level. Still, the number of cells displaying detectable TEM activity was strongly reduced upon infection with the LVSΔ*iglG* mutant compared to WT LVS (S12 Fig) suggesting that IglG is required for optimal Type VI secretion in macrophages infected with LVS.

**Fig 9:**
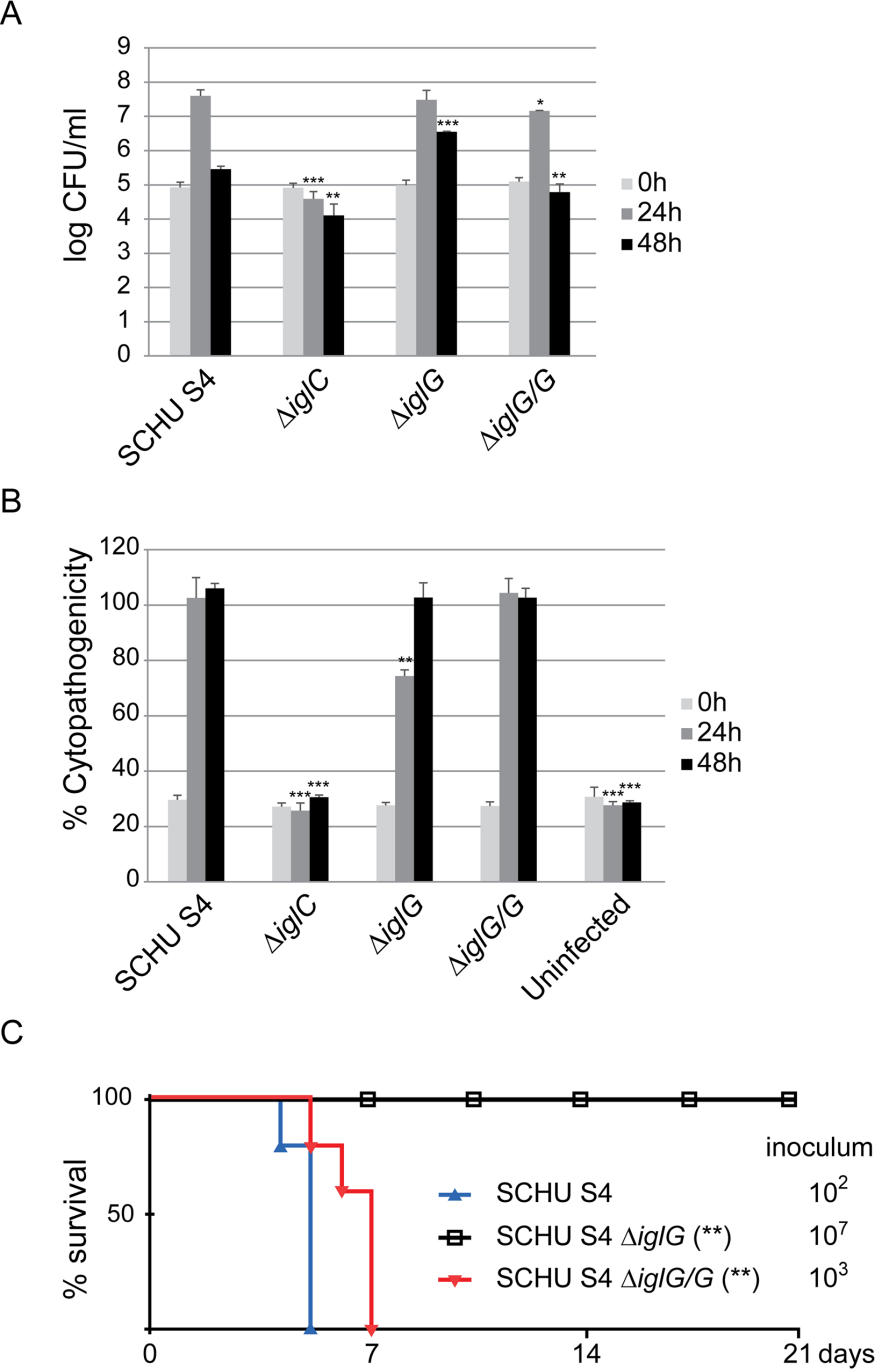
IglG is required for SCHU S4 virulence *in vivo* but is dispensable for *in vitro* replication and cytopathogenicity. Intracellular growth and cytopathogenicity in J744 cells (A and B, respectively) of *F*. *tularensis* subsp. *tularensis* strains. (A) J774 cells were infected at an MOI of 200 for 2 h. Upon gentamicin treatment, cells were allowed to recover for 30 min after which they were lysed immediately (corresponds to 0 h) or after 24 or 48 h with PBS-buffered 0.1% sodium deoxycholate solution and plated to determine the number of viable bacteria (log_10_). Infections were repeated twice and a representative experiment is shown. Each bar represents the mean values and the error bar indicates the SD from triplicate data sets. The asterisks indicate that the log_10_ number of CFU was significantly different from the parental SCHU S4 strain as determined by a 2-sided *t*-test with equal variance (*, *P* ≤ 0.05; **, *P* ≤ 0.01; ***, *P* ≤ 0.001). (B) Culture supernatants of infected J774 cells were assayed for LDH activity at 0, 24 and 48 h and the activity was expressed as a percentage of the level of non-infected lysed cells (positive lysis control). Values of triplicate wells (means +/- SD) from one representative experiment of two are shown. The asterisks indicate that the cytopathogenicity levels were different from those of SCHU S4-infected cells at a given time point as determined by a 2-sided *t*-test with equal variance (**, *P* ≤ 0.01; ***, *P* ≤ 0.001). (C) Survival of mice following intradermal challenge with SCHU S4 strains. Groups of five mice were challenged with the indicated inoculum and monitored for 21 days for signs of illness. (A-B) Each bar represents the mean values and the error bar indicates the SD from triplicate data sets. (C) Mantel-Cox test was performed; *P*-values for the comparison with WT survival curves are shown.

The role of IglG in highly virulent *F*. *tularensis* had until now not been investigated. We therefore generated a SCHU S4 Δ*iglG* mutant by deletion of the two *iglG* gene copies and assessed its ability to grow within macrophages as well as to cause cytotoxicity. In J774 cells, the phenotype was very similar to that of LVS Δ*iglG* in that the mutant exhibited no detectable growth defect (Fig 9A) [25], and it showed an intermediate cytotoxic effect, although with a slightly faster kinetics compared to that of the LVS Δ*iglG* mutant (compare Figs 8A and 9B). Thus, the SCHU S4 Δ*iglG* mutant exhibited an intermediate cytotoxic effect at 24 h, but not at 48 h, which could be restored to wild-type levels by expressing LVS-derived IglG (Fig 9B). The extensive cell death observed in SCHU S4-infected cells was likely responsible for the drop in bacterial counts observed between 24h and 48h. Accordingly, the delayed cytotoxicity kinetics observed upon infection with the Δ*iglG* mutant correlated with a lower decrease in bacterial counts between 24h and 48 h vs. SCHU S4 (Fig 9A and 9B). As expected, the Δ*iglC* mutant was severely defective for both growth and cytotoxicity (Fig 9A and 9B). We also confirmed that *F*. *novicida*-derived IglG was equally efficient as that of LVS in restoring the defective cytopathogenic response of the SCHU S4 Δ*iglG* mutant in J774 cells, while the *F*. *novicida* mutant variants that either lacked the two α-helices of the N-terminal extension (IglG_Δ2–39_) or carried a C64G substitution were unable to complement the mutant. We then analyzed the virulence of the SCHU S4 Δ*iglG* mutant in a mouse model of tularemia. The mutant was avirulent in mice, since it did not cause any mortality even when 10^7^ cfu of the mutant were inoculated intradermally (Fig 9C). As expected, both the WT strain and the complemented Δ*iglG* mutant killed 100% of the mice at much lower inoculum (10^2^ and 10^3^, respectively). Taken together, this comparison validates the role of the two identified IglG domains for *F*. *tularensis* T6SS function and virulence.

### IglG interacts with IglF in an N-terminal α-helix-dependent manner

**Fig 10:**
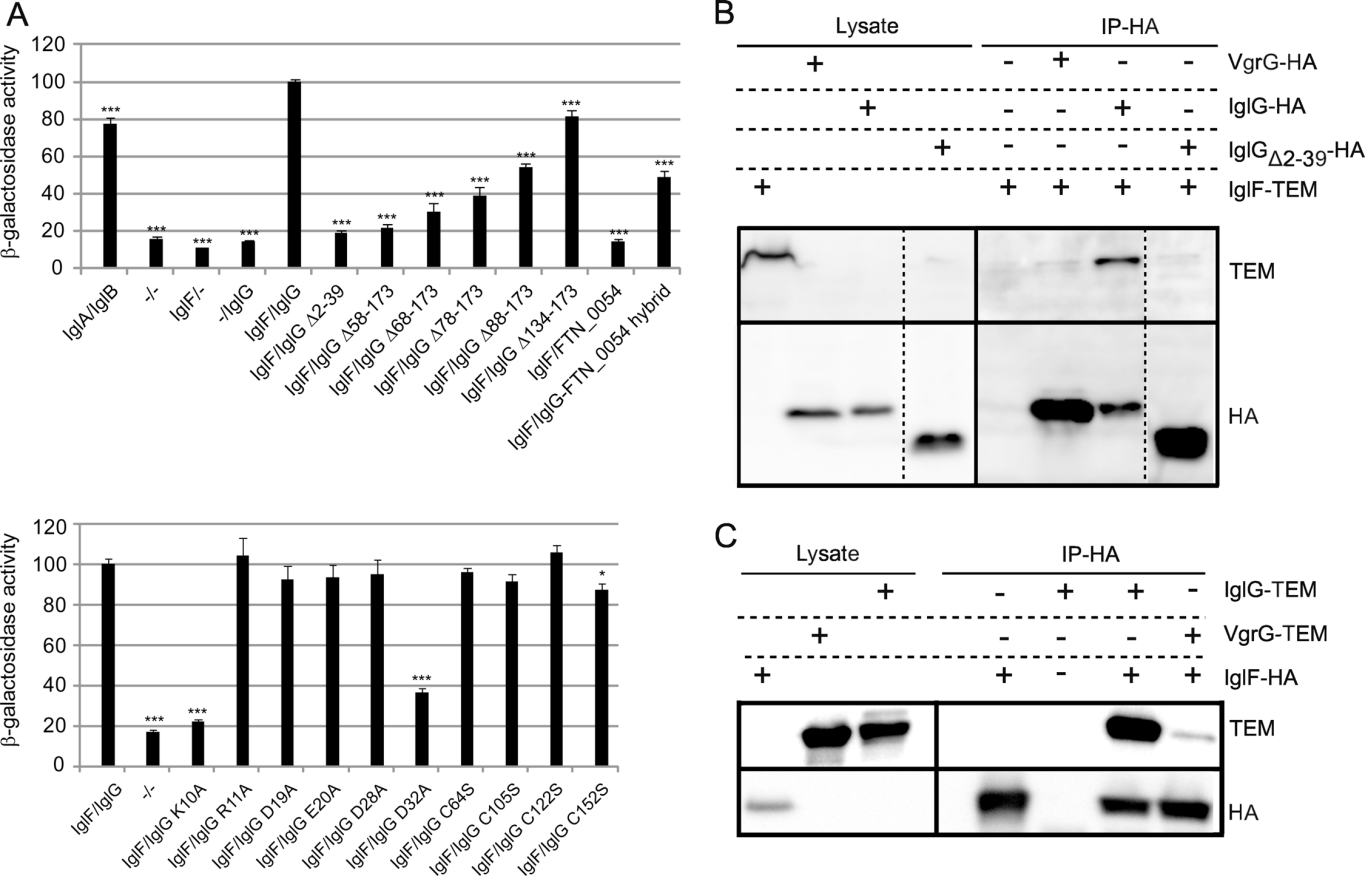
IglG interacts with IglF in an N-terminal extension (AA 1–39)-dependent manner. (A) A B2H analysis of IglF and IglG, when fused to Zif and to the ω subunit of *E*. *coli* RNAP respectively, induces transcription from the *lacZ* promoter of the *E*. *coli* reporter strain KDZif1ΔZ, resulting in β-galactosidase activity. The ability of mutant forms of IglG to interact with IglF was analyzed and the levels of interaction expressed as percentage of the wild-type interaction, which was set to 100%. The negative controls correspond to vectors missing inserts, while the positive control is IglA-IglB in the upper panel and IglF-IglG in the lower panel [43]. Shown is the percentage of mean β-galactosidase activity ± SEM from four independent experiments, where two to three independent transformants were tested on each occasion. Unpaired *t*-tests were performed to compare the β-galactosidase activity obtained with the indicated mutant IglG protein to that of wild-type IglG (*, *P* ≤ 0.05; ***, *P* ≤ 0.001). (B, C) Lysates from *F*. *novicida* expressing 2HA-tagged VgrG, IglG, IglG_Δ2–39_ (B) or IglF (C) proteins were incubated with anti-HA agarose beads. The loaded beads were then incubated with lysates from *F*. *novicida* expressing or not TEM-tagged IglF (B) IglG or VgrG (C) and used for co-immunoprecipitation. Proteins in bacterial lysates and immunoprecipitated proteins were revealed by immunoblotting using specific antibodies against the HA- or TEM tags.

N- and C-terminal extensions of PAAR proteins possess either enzymatic activities or are cargo domains enabling effector secretion [16,42]. To test whether the IglG N-terminal extension could act as such a cargo domain, we performed a bacterial two-hybrid (B2H) interaction screen for IglG against all other FPI proteins. We identified a specific interaction between IglG and IglF, the latter encoded by the gene immediately upstream of *iglG* (Fig 10A upper panel and S1 Fig). This interaction was validated by co-immunoprecipitation in *F*. *novicida* (Fig 10B and 10C). To identify the IglF-interacting domain within IglG, we generated specific mutations within the latter protein and tested them in the B2H system. The amount of β-galactosidase activity, indicative of a stronger interaction, increased gradually when smaller truncations were introduced in the IglG protein (Fig 10A, upper panel). Strikingly, the interaction was completely abolished when the first 39 residues of IglG were removed (IglG_Δ2–39_) (Fig 10A, upper panel). This result was validated by co-immunoprecipitation (Fig 10B). In contrast, deletion of the last 39 of the protein (IglG_Δ134–173_) had only a minor effect on the interaction (Fig 10A, upper panel). In agreement with these results, we did not observe any interaction between IglF and the other *F*. *novicida* PAAR-like protein FTN_0054, which lacks an N-terminal extension. While the N-terminal extension was clearly essential for the interaction, it was not sufficient in this system, since the N-terminal extension alone (IglG_Δ58–173_) did not interact with IglF (Fig 10A, upper panel). This lack of interaction may be partly due to improper folding of the truncated protein. In support of this hypothesis, a protein consisting of the first 60 residues of IglG fused to the PAAR-like protein FTN_0054 (starting at the shared alanine at position 10, see Fig 1A) established an interaction with IglF, albeit partially reduced (Fig 10A, upper panel). To identify key residues that may participate in the binding, a helical wheel analysis of the IglG N-terminus was performed using the first 56 residues in the analysis. Charged residues, both positively and negatively, at the different sides of the predicted helix were targeted for alanine scanning resulting in single substitutions K10A, R11A, D19A, E20A, D28A and D32A. Strikingly, when tested in the B2H assay, two of these substitions either abolished (K10A) or severely diminished (D32A) binding to IglF (Fig 10A, lower panel). Interestingly, these residues were predicted to lie close to each other in the same side of the helix. Importantly, individual substitutions of each of the single cysteines to either serine or alanine had no effect on the interaction (Fig 10A) further suggesting that the PAAR-like domain *per se* and the metal-binding ability are not essential for the interaction with IglF. Altogether our results indicate that IglG harbors a PAAR-like domain and an N-terminal extension, which interacts with IglF. The N-terminal extension is required for virulence, possibly leading to the translocation of this putative effector protein into the host cell.

## Discussion

Despite the well-recognized role of the FPI in *Francisella* virulence *in vitro* and *in vivo*, the function of most of the FPI-encoded proteins is still elusive. The homology between FPI proteins and components of a T6SS [44,45] was described almost 10 years ago [8]. Yet, in spite of tremendous progress in the understanding of T6SS assembly and function in numerous bacterial species [11,14], the translation of this knowledge to the *Francisella* FPI has been challenging. This difficulty lies in part in the limited similarity of the *Francisella* FPI with other T6SS [46].

In this work, we focused on IglG, a protein required for the virulence in mice of *F*. *novicida*, *F*. *tularensis* LVS and of the highly virulent *F*. *tularensis* SCHU S4 strain ([7], this work). We identified IglG as a protein containing a DUF4280 domain. This work and another recent study [28] identified IglG and two other DUF4280-containing proteins (FTN_0054 and Fjoh_3275/FteI) as three proteins likely to adopt a tertiary structure homologous to that of PAAR proteins. In addition, we found that the DUF4280 consensus sequence itself was also predicted to adopt a PAAR-like fold suggesting that the results we obtained on IglG will be valid for most of the DUF4280 proteins present in a large number of bacterial species. We thus propose to assign the term PAAR-like domain to this domain of unknown function, characterized by four highly conserved cysteines. Although we cannot completely rule out that the cysteines might form disulfide bridges, our biochemical analyses and the predicted homology between the zinc-binding PAAR protein and the PAAR-like proteins strongly suggests that the structural fold of PAAR-like proteins is stabilized by the cysteine-mediated metal coordination. Accordingly, we identified IglG as a metal-binding protein. *In vitro*, IglG had a small preference for iron over zinc in contrast to the typical PAAR protein PA_0824 from *P*. *aeruginosa*, which in our experimental conditions, preferentially binds zinc (S7 Fig). Many metalloproteins have the potential to bind different metal ions. The binding of a specific divalent cation to a protein is controlled by the natural order of stability of complexes of bivalent transition metals (also known as the Irving-Williams series: Mg^2+^ and Ca^2+^ (weakest binding)< Mn^2+^ < Fe^2+^ < Co^2+^ < Ni^2+^ < Cu^2+^ > Zn^2+^), the relative concentrations of the different cations as well as specific mechanisms, such as the presence of metallochaperones and chelators [47,48]. Indeed, we have observed that purified IglG can bind different cations *in vitro* (Fig 4C). The nature of the metal contained within IglG protein when expressed at physiological level in *Francisella* within its mammalian host may be influenced by the localization of the protein and the relative concentrations of the different divalent cations in each compartment as well as by specific mechanisms such as the presence of metallochaperones. Furthermore, innate immune mechanisms such as Zn^2+^ chelation by calprotectin, one of the most abundant protein of the neutrophil cytosol [49] may influence the bioavailability of divalent cations and the nature of the IglG-bound metal. Mutation of single cysteine residues only moderately decreased metal binding, suggesting that three functional side chains are sufficient to bind metal, a feature that has been observed in other proteins [50–52]. However, *in vivo*, each individual cysteine is required to sustain the role of IglG in *F*. *novicida* virulence suggesting that tetravalent coordination of the metal is required for IglG stability and function. Interestingly, the tip of the membrane-attacking complex of bacteriophages P2 and φ92, which is homologous to the VgrG_3_-PAAR complex of the T6SS binds iron [53,54]. These results highlight the evolutionary conservation of spike proteins and identify PAAR-like proteins as potential evolutionary intermediates between phage spike proteins and bacterial T6SS PAAR proteins.

The predicted PAAR-like fold suggests that PAAR-like proteins are located at the tip of the T6SS. Despite its very small size, *Francisella* VgrG displays a β-structural repeat consistent with an ability to bind PAAR-like proteins [16] although we failed to experimentally demonstrate an interaction between IglG and VgrG by both bacterial two-hybrid and pull-down experiments. Strikingly, while the deletion of the PAAR-like gene *iglG* confers to *F*. *novicida* a phenotype identical to the one observed after the deletion of the *hcp* homologue *iglC*, this is not true in *F*. *tularensis* LVS and *F*. *tularensis* SCHU S4 strain. These results suggest that in *F*. *tularensis*, the activity of the T6SS is less stringently dependent on the capping of the T6SS by a PAAR-like protein. Accordingly, using TEM fusion proteins and a β-lactamase/CCF4 flow cytometry assay, we observed a significant (although strongly reduced compared to WT LVS) secretion of both IglC and VgrG in the LVS Δ*iglG* mutant. IglG may thus play an auxiliary role in *F*. *tularensis* T6SS function. Yet, it is clearly required for *F*. *tularensis* virulence *in vivo* ([7], this work) highlighting the importance of a fully functional T6SS to trigger disease.

As described above, another PAAR-like protein (FTN_0054) is found in the *Francisella novicida* island (FNI). We could not find evidence of this genomic island playing any major role in the virulence of *F*. *novicida in vitro* nor *in vivo* in a mouse model of tularemia (S14 Fig). It remains to be demonstrated whether this island encodes a functional T6SS involved in bacterial competition and/or in the targeting of specific eukaryotic cells. Interestingly, one FNI protein (FTN_0052) exhibits similarity to proteins from the phosphoesterase family (pfam04185), which includes both bacterial phospholipase C enzymes and eukaryotic phosphatases making this protein a likely T6SS effector [55]. FTN_0052 has no homologue in the FPI, suggesting that the two genomic islands may encode different arrays of effectors. On the other hand, unique features of the FPI include the N-terminal extension of the PAAR-like protein IglG (compared to FTN_0054) as well as IglF. The comparison of the FPI and the FNI might thus help us to discriminate the proteins involved in the T6SS machinery *sensus stricto* from specific proteins associated with virulence towards the mammalian host. Interestingly, the *iglF* gene lies in between the *vgrG* and the *iglG* genes. This specific genetic linkage supports our experimental data demonstrating the key role of the IglG N-terminal extension in establishing an interaction with IglF.

**Fig 11:**
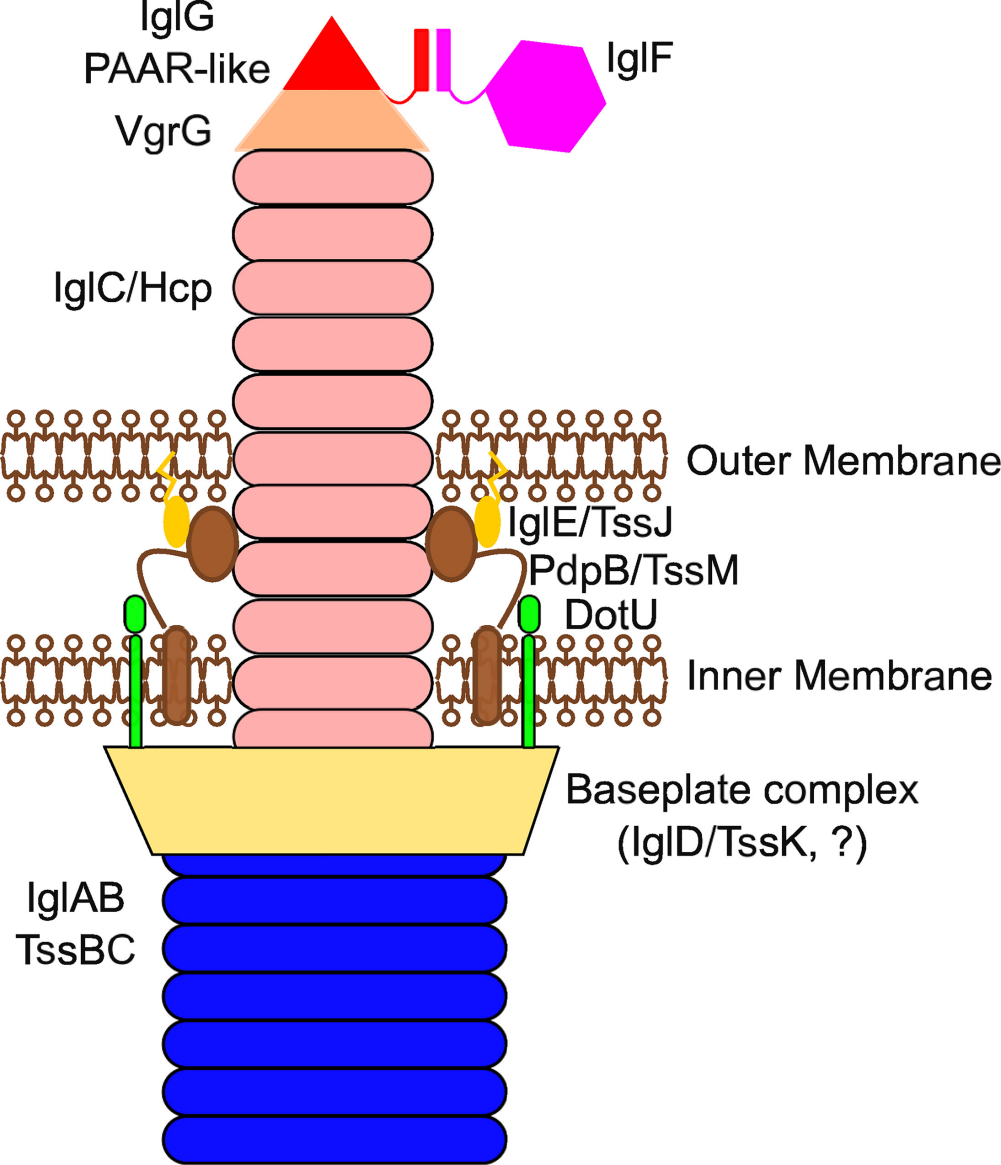
Putative model for the *Francisella* T6SS. The model was adapted from [13]. The *Francisella* T6SS is presented in the contraction phase. While the interaction of IglF and IglG was demonstrated in this study, the function and the localization of IglF within the T6SS remain to be determined. While the current knowledge on VgrG and PAAR proteins suggests that the two *Francisella* homologues may be located at the tip of the T6SS, this remains to be demonstrated.

PAAR-like domains are frequently extended in an N- or C-terminal manner by domains with known T6SS effector functions (Fig 6), strengthening the functional homology between PAAR [16] and PAAR-like proteins. The N-terminal extension that we identified in IglG is predicted to protrude outside of the PAAR-like core domain (Fig 1B and S10 Fig) suggesting it might act as an adaptor domain to connect the tip of the T6SS with other T6SS proteins/effectors. The adaptor nature of the N-terminal extension of IglG is supported by the fact that IglG interacts with IglF in a manner that requires the first 39 residues of IglG. The same region within IglG is dispensable for *in vitro* secretion of IglG, while the PAAR-like domain is required for IglG secretion. In line with identified PAAR-protein functions [16], these data suggest that IglG could act as a cargo molecule aiding in the T6SS-mediated delivery of IglF. While deciphering the role of IglF in the T6S machinery or in infected cells will be the goal of future studies, IglF is a secreted protein [24] required for *in vitro* replication [21], suggesting that IglF may be a *Francisella* effector secreted into the host cell in an IglG-dependent manner. Altogether, this work in addition to increasing our understanding of the structure of *F*. *tularensis* T6SS (see model in Fig 11) identifies a novel family of PAAR-like proteins with conserved cysteines and metal binding ability, involved in connecting the Type VI secretion machinery to effector activities.

## Materials and Methods

### Ethics statement

All experiments involving animals were reviewed and approved by the animal ethics committees of the University of Lyon, France under the protocol numbers #ENS_2014_017 and #ENS_2012_061, by the IRB of the National Research Council, Ottawa, Canada and by the Local Ethical Committee on Laboratory Animals, Umeå, Sweden (no. A67-14).

### Mouse infections

For testing of SCHU S4 and derivatives, BALB/c mice were purchased from Charles River Laboratories (St. Constant, Quebec, Canada). The mice were maintained and used in accordance with the recommendations of the Canadian Council on Animal Care Guide to the Care and Use of Experimental Animals in a federally licensed, Select Agent-approved, small animal containment level 3 facility, National Research Council, Ottawa, Canada. *F*. *tularensis* strains were injected in a volume of 50 μl intradermally in groups of five (n = 5). The mice were examined daily for signs of infection and were euthanized by CO_2_ asphyxiation as soon as they displayed signs of irreversible morbidity. For testing of U112 and derivatives, C57BL/6J mice were injected in a volume of 100 μl intradermally in groups of five (n = 5). Aliquots of the diluted cultures were also plated on GC-agar to determine the number of CFU injected. Actual doses were the following: 830 (U112), 915 (Δ*iglG*), 1,635 (Δ*iglG*/IglG-2HA), 1,065 (Δ*iglG*/IglG_Δ2-39_-2HA) and 600 (Δ*iglG*/IglG_C64G_-2HA). Previous studies have demonstrated that the LD_50_ of the U112 strain is approximately 500 CFU [56]. Mice were examined twice daily for signs of severe infection and euthanized by CO_2_ asphyxiation as soon as they displayed signs of irreversible morbidity. In our experience, such mice were at most 24 h from death, and time to death of these animals was estimated on this premise.

### Bacterial strains and targeted mutagenesis

Bacterial strains used in this study are listed in S1 Table. *E*. *coli* strains were cultured in Luria Bertani broth (LB) or on Luria agar plates at 37°C. *F*. *tularensis* strains were grown on modified GC-agar base at 37°C. *F*. *novicida* strains were grown in tryptic soy broth (TSB) supplemented with 0.1% (w/v) cysteine. When applicable, carbenicillin (Cb; 100 μg/ml), tetracycline (Tet; 10 μg/ml), kanamycin (Km; 50 μg/ml for *E*. *coli*, 10 μg/ml for *F*. *tularensis*), or chloramphenicol (Cm; 25 μg/ml for *E*. *coli*, 2.5 μg/ml for *F*. *tularensis*) were used.

*F*. *novicida* chromosomal deletion mutants of *iglG*, *vgrG* or *FTN_0037-FTN_0054* (FNI) locus were constructed as previously described [57] by allelic exchange using PCR products followed by Flp-mediated excision of the antibiotic-resistance marker [58] or by using the suicide vector pJEB753 [59]. Primers are presented in S2 Table. To construct the Δ*iglG* deletion mutant in SCHU S4, the suicide vector pJEB866 was used. pJEB866 was constructed by lifting the deletion fragment from pJEB753 into pDMK2 [60] using *Xho*I/*Sac*I digestion. Conjugal mating experiments using S17-1λ pir as the donor strain and sucrose-selection allowed for the allelic exchange of the suicide plasmids within regions of complementary sequence on the chromosome of U112 or SCHU S4 [3]. To remove both copies of the *iglG* gene in the latter strain, the procedure was repeated. PCR screening and genomic sequencing were used to verify that the anticipated genetic event had occurred.

### Construction of expression vectors

Plasmids used in this study are listed in S1 Table. Primer combinations and restriction sites used to generate the plasmids are listed in S2 Table. All amplified fragments were first cloned into pCR4-TOPO TA cloning vector to facilitate sequencing. PCR or overlap PCR was used to introduce substitution or deletion mutations within *iglG*. A hybrid gene consisting of IglG and FTN_0054 was constructed using LVS and U112, respectively as template in the first PCR reaction step. Upon overlap PCR, the resulting hybrid consisted of the first 60 residues of IglG, and where the remaining part was derived from FTN_0054, starting at alanine at position 10. 2xHA-tagged IglG was generated by cloning *iglG* in frame with a single HA tag sequence in the popHA plasmid (kindly provided by P. Mangeot) followed by addition of a second HA tag sequence by PCR amplification. Plasmids used for complementation of Δ*iglG* in *trans* were constructed by introducing C-terminally 6xHis-tagged or 2xHA-tagged wild-type or mutated versions of *iglG* into the *Nde*I/*Eco*RI sites of pKK289Km [61] or into a cyaA-containing pFNLTP6 derivative [62,63] respectively. The latter plasmid was also used to create TEM fusion after removal of the plasmid encoded β-lactamase gene. *E*. *coli* TEM-1 amplified from pUC19 (ThermoFisher Scientific) was then cloned under the Gro promoter using *Nhe*I/*Bam*HI sites. FPI and controls genes were cloned in frame with TEM-1 using *Eco*RI and *Nhe*I sites. Plasmids used for B2H analysis were constructed by introducing *Nde*I/*Not*I fragments of mutated *iglG* into pBRGPω [64]. For GST-IglG expression, the *F*. *novicida iglG* gene was PCR-amplified and cloned into the pGEX-6-P3 (Novagen) in frame with the GST-encoding gene. Plasmids were transferred into bacteria by chemical transformation or electroporation.

### Bacterial 2-hybrid analysis

*E*. *coli* strain KDZif1ΔZ was used as the reporter strain for the bacterial-2-hybrid experiments. It harbors an F9 episome containing the lac promoter-derivative placZif1–61 driving expression of a linked lacZ reporter gene [64]. Cells were grown with aeration at 37°C in LB supplemented with 1 mM IPTG (Isopropyl β-D-1-thiogalactopyranoside). Cells were permeabilized with SDS-CHCl3 and assayed for β-galactosidase (β-gal) activity as described previously [65].

### Cell lines and bone marrow-derived macrophages

Preparation and culture of bone marrow macrophages (BMDMs) were performed as previously described [36]. All mice were in the C57BL/6J background (Charles River, France). J774A.1 macrophage-like cells (Cellulonet, Lyon, France) [66] and L929 ISRE-luciferase cells (obtained from B. Beutler, The Scripps Research Institute) [67] were cultured in DMEM (ThermoFisher Scientific) supplemented with 10% fetal calf serum (Lonza) and 1mM glutamine.

### Infections and replication assay

BMDM were infected as described before [68] at the indicated multiplicity of infection (MOI). For *F*. *novicida* intracellular replication assay, macrophages were lysed with 1% (w/v) saponin (Sigma) in water for 5 min. Dilution, plating on TSA supplemented with 0.1% (w/v) cysteine and counting was performed using the easySpirale Dilute (Interscience). For LVS and SCHU S4, cells were infected for 2 h at an MOI of 200, washed three times, and incubated in the presence of gentamicin (5 μg/ml; LVS and 2 μg/ml; SCHU S4) for 30 min (corresponds to time zero). At 0, 24 and 48 h, the macrophage monolayers were lysed in PBS with 0.1% deoxycholate, serially diluted in PBS and plated on modified GC-agar base plates for determination of viable counts.

### Interferon and TNF-α secretion

Type I interferon secretion was determined by an ISRE-luciferase bioassay [67]. L929 ISRE-luciferase cells were plated the day before at 10^5^ cells per well in a 96 wells-plate. Supernatants from infected BMDMs were added for 4 h onto the ISRE-luciferase cells. Luciferase luminescence was detected using Bright Glo Assay (Promega) following the manufacturer’s instructions. TNF-α secretion of J774 cells upon 2 h of LPS stimulation was performed according to our previously established protocols [7], using the BD OptEIA Mouse TNF-α ELISA Set (BD Biosciences) according to the manufacturer’s instructions.

### Cell death assays

Cell death was monitored in BMDMs by monitoring in real time incorporation of propidium iodide (used at 5 μg/ml) through measurement of fluorescence emission at 635 nm every 15 min on a microplate reader (Tecan). Quantification of cytopathogenicity in J774 cells was performed by analysis of LDH release in the cell supernatant, using the CytoTox96 LDH kit (Promega, France), following manufacturer’s instructions.

### Phagosomal rupture assay

Quantification of vacuolar escape using the β-lactamase/CCF4 assay (Life technologies) was performed as previously described [33]. Briefly, bone marrow-derived macrophages seeded onto non-treated plates were infected as described above for 2 h, washed and incubated in CCF4 for 1 h at room temperature in the presence of 2.5 mM probenicid (Sigma). Propidium iodide negative cells were considered for the quantification of cells containing cytosolic *F*. *novicida* using excitation at 405 nm and detection at 450 nm (cleaved CCF4) or 510 nm (intact CCF4).

### Bioinformatic analysis

Identification of conserved domains, of the DUF4280 consensus sequence and of the species presenting DUF4280 proteins were performed using CD database, the Conserved Domain Architecture Retrieval Tool (CDART) [69] and the Interpro database. Three dimensional modeling was performed using the default parameters in the I-Tasser and Phyre-2 webservers [30]. Five models were obtained for each template (DUF4280 and IglG) with very high scores. Figures of structure were generated with Pymol (Delano Scientific, https://www.pymol.org). Conserved residues were identified using the iterative search algorithm Jackhmmer [70]. The N-terminal α-helix was analysed using EMBOSS::pepwheel software (http://www.tcdb.org/progs/?tool=pepwheel). For the molecular dynamics protocol, IglG was modeled with the AMBER99SB force field [71]. The model was ionized and solvated with TIP3P water molecules, setting unit cell dimensions to 61 Å × 57 Å × 73 Å (residues 53–173). The resulting ~24000 atoms system was minimized and equilibrated locally with ACEMD [72] for 2 ns under NPT conditions, of 1 atm at 300 K, nonbonded cutoff of 9 Å, rigid bonds, and PME electrostatics. A time step of 4 fs was used, in conjunction with a hydrogen mass-repartitioning scheme. During minimization and the first 2 ns of equilibration, the protein’s heavy atoms were restrained by a harmonic potential with k = 1 kcal mol^−1^ Å^−2^. The complete molecular dynamics resulted in a 400ns trajectory where the rmsf was computed by means of the Gromacs subroutines [73].

### Recombinant IglG production and purification

For protein expression, GST, GST-Igl*G*, His_6_-IglG encoding vectors were introduced into the *E*. *coli* Rosetta strain, which was cultured at 37°C in LB medium supplemented with 100 μg/ml ampicillin to an optical density at 600 nm of 0.8–1.0. Protein expression was induced by adding 1 mM IPTG at 20°C for 16 h. Cells were harvested by centrifugation and re-suspended in lysis buffer (20 mM Tris pH 8.0, 0.5 M NaCl, 10% Glycerol (V/V), 1% triton (V/V) supplemented with 1 mg/ml of lysozyme (Sigma), DNAse (Sigma) and Ethylenediamine tetra-acetic acid (EDTA)-Free protease inhibitors cocktail (Roche)). Cells were disrupted by sonication and the resulting lysate was cleared by centrifugation (20 min at 16,000 x g at 4°C). For GST and GST-IglG, the supernatant was loaded onto a GST-HiTrap column (GE Healthcare), equilibrated with 15 ml of buffer (20 mM Tris pH 8.0, 300 mM NaCl, 5% glycerol). The column was washed with 20 ml of buffer containing 20 mM Tris pH 8.0, 300 mM NaCl, 5% glycerol (V/V). The proteins were eluted with 10mM glutathione and concentrated using Amicon 0.5 mL 10K (Millipore) centrifugal devices. The proteins were dialysed against 20 mM Tris pH 8.0, 300 mM NaCl, 5% Glycerol (V/V) at 4°C. To reach more than 95% purity (as assessed by sodium dodecyl sulphate–polyacrylamide-gel electrophoresis), the proteins were further purified by gel filtration using the Superdex 75 column or by the anion exchange monoQ hiTrap Q HP column (GE Healthcare). His_6_-IglG protein was extracted from the insoluble fraction. Following lysate centrifugation, the pellet was re-suspended in 8M Urea buffer, 20mM Tris pH8 under strong agitation during 20 minutes at room temperature. The resulting solubilized proteins were cleared by centrifugation (15 min at 10,000 x g at 4°C). Supernatant was collected and loaded onto a 5 ml His-trap column (GE Healthcare). The column was washed once with 10mL buffer containing 8M Urea buffer, 20mM Tris pH8, 1M NaCl, once with buffer containing 25 mM imidazole. The protein was eluted with a linear gradient of imidazole (25–500 mM) in Urea 8M, 20mM Tris pH8. His_6_-IglG was refolded by dialysis overnight in buffer containing 20mM Tris pH8, 250mM arginine, 100mM NaCl, 5% glycerol, 1mM EDTA. Arginine was eliminated by three sequential 2 hours dialysis at 4°C, the first one in buffer containing 20mM arginine, 20mM Tris pH8, 300mM NaCl, 5% glycerol, 1mM EDTA, the second and the third ones in 20mM Tris pH8, 300mM NaCl, 5% glycerol, 1mM EDTA. Protein concentrations were determined by measuring the UV light absorbance at 280nm.

### Metal concentration determination by inductively coupled plasma mass spectrometry (ICP-MS)

100 μl of each sample was diluted to 10 ml in 2% HNO_3_ with Indium as an internal standard. Zn, Fe, Ni, Cu, Pb and In were measured by ICP-MS (iCAP Q ThermoFisher Scientific) with running conditions presented in S3 Table.

### Circular dichroism (CD) spectroscopy and IglG-metal binding

Far-UV CD spectra (190nm-260nm) of His_6_-IglG were recorded on a Chirascan Circular Dichroism Spectrometer (Applied photophysics) calibrated with (1S)-(+)-10-camphorsulfonic acid. Measurements were carried out at room temperature in a 0.1cm path length quartz cuvette. Parameters were set as followed: wavelength range 180-260nm, 0.2nm increment, bandwith 0.5nm; scan speed, 50 nm.min^-1^; response time 1s. Spectra were corrected by subtracting buffer contributions and protein dilution factors before calculating the mean residue molar ellipticity. Spectra deconvolution was performed using Dichroweb server [74] and the Selcon3 algorithm [75] on His_6_-IglG refolded in the presence of FeCl_2_ at 1mM and dialyzed four times in a buffer consisting of NaH_2_PO4 20mM, NaF 300mM, pH 7.5. To follow metal binding by CD spectrometry, His_6_-IglG in Tris 20mM pH8, NaCl 200mM, glycerol 5% was diluted to 10 μM in NaH_2_PO4 20mM pH 7.5, NaF 300mM, glycerol 5% buffer. Spectra were measured immediately after dilution or following addition of increasing amount of FeSO_4_, ZnSO_4_, MgSO_4_, CoSO_4_ from 0.2 to 2 molar equivalent of protein. TCEP was incubated with His_6_-IglG at 0.5, 5 and 50 molar equivalent of protein for up to 4 hours on ice before spectra acquisition. Spectra were smoothed using Prism software.

### Co-immunoprecipitation

*F*. *novicida* expressing 2xHA- or TEM1-tagged proteins were subcultured to an O.D._600_ of 1.5 and lysed by sonication in Tris 50mM, NaCl 150mM buffer containing an EDTA-free protease inhibitor cocktail (Roche). HA_2_-tagged proteins expressing lysates (equivalent to 4 ml of bacterial culture) or 2% BSA in Tris 50mM, NaCl 50mM as a control were incubated with 10μl of anti-HA agarose antibody (Sigma) slurry for 2h at 4°C. Beads were washed four times with the same buffer and incubated for 3h at 4°C with TEM1-tagged proteins expressing lysates (equivalent to 4 ml of bacterial culture) under mild agitation. Beads were then washed twice with Tris 50mM, NaCl 50mM, NP40 1% and twice with Tris 50mM, NaCl 50mM before being boiled in Laemmli sample buffer (Tris-HCl 50mM, SDS 1%, glycerol 5%, Bromophenol blue 0.0005%) with β-mercaptoethanol (286 μM).

### Immunoblotting analysis

Protein lysates for immunoblotting were prepared by using Laemmli sample buffer. Protein lysates corresponding to equal OD_600_ were loaded on 4–12% Bis/Tris gels (Invitrogen), and run in TGS buffer. Protein transfer was performed with iBlot gel transfer stacks (Invitrogen). Membranes were probed with the following monoclonal antibodies: mouse anti-PdpB, mouse anti-IglC, and mouse anti-IglB (all provided from BEI Resources, Manassas, VA, USA) or via commercially available anti-HA (clone HA-7, Sigma), anti-Penta-His (Qiagen, MD, USA). A secondary horseradish peroxidase (HRP)-conjugated goat anti-mouse antibody (Santa Cruz Biotechnology, CA, USA) and the Enhanced Chemiluminescence system (ECL) (Amersham Biosciences, Uppsala, Sweden) were used.

### Secretion assays

Protein secretion during infection was estimated using TEM-β-lactamase fusion proteins and CCF4 substrate as previously described [24] by flow cytometry analysis using a Canto II analyser (BD). The in *vitro* KCl assay was performed as previously described [23]. Briefly, *F*. *novicida* was grown overnight in TSB containing 0.1% cysteine. The overnight culture was diluted to OD_600_ = 0.3 in medium with or without 5% KCl. Upon reaching an OD_600_ = 1.5, the culture was centrifuged at 4,700 x g for 20 min at 4°C. The supernatant was filtered-sterilized using a 0.22 μm filter and proteins were extracted by methanol-chloroform precipitation. Protein extracts corresponding to 0.045 and 1 OD_600_ of the pellet and the concentrated supernatant fraction, respectively were loaded onto the SDS-PAGE gel and immunoblotted with the indicated antibodies.

### Statistical analysis

Statistical data analysis was performed using unpaired *t*-tests and Prism 5.0a software (GraphPad Software, Inc.). Two-tailed *P*-values are shown. For survival experiments, Log-rank (Mantel-Cox) test were performed. The following convention is used: *, *P* ≤ 0.05; **, *P* ≤ 0.01; ***, *P* ≤ 0.001.

### Accession numbers

For each strain, when applicable, the discontinued NCBI gene record and the current NCBI locus tag are indicated. For *F*. *tularensis* strains, the two FPI loci are indicated. NA: Not applicable.

VgrG: U112; FTN_1312; FTN_RS06720; LVS; FTL_0123; FTL_RS00610; FTL_1169; FTL_RS05950; SCHU S4; FTT_1702; FTT_1347

IglC: U112; FTN_1322; FTN_RS06770; LVS; FTL_0113; FTL_RS00560; FTL_1159; FTL_RS05900; SCHU S4; FTT_1712; FTT_1357

IglG: U112; FTN_1314; FTN_RS06730; LVS; FTL_0121; FTL_RS00600; FTL_1167; FTL_RS05940; SCHU S4; FTT_1704; FTT_1349

IglF: U112; FTN_1313; FTN_RS06725; LVS; FTL_0122; FTL_RS00605; FTL_1168; FTL_RS05945; SCHU S4; FTT_1703; FTT_1348

FTN_0054: U112; FTN_0054; FTN_RS00280; LVS; NA; SCHU S4; NA

FTN_0052: U112; FTN_0052; FTN_RS00270; LVS; NA; SCHU S4; NA

*P*. *aeruginosa* (strain PAO1): PA_0824

## Supporting Information

S1 Fig Schematic structures of the *Francisella* Pathogenicity Island (FPI) and of the *Francisella novicida* island (FNI). Genomic organizations of the FPI (FTN_1309-FTN_1325) and of the FNI (FTN_0037–0054) are shown. For clarity purposes, the ORFs were not drawn to scale. Encoded proteins homologous to canonical T6SS components are indicated using the Tss nomenclature [17]. ORFs unique to only one of the *F*. *novicida* genomic islands and with no Tss homologue are colored in white. The FNI encodes a putative T6SS [10,28,76] with a DUF4280-containing protein (FTN_0054) lacking an N-terminal extension. IglG and FTN_0054 are indicated by arrows. The encoded proteins investigated in this study are indicated in bold font.
(EPS)

S2 Fig A molecular dynamics simulation with a 400 ns trajectory suggests that the predicted PAAR-like structure of IglG would be stable over time. The root mean square fluctuation (RMSF) as a function of residue number of IglG C-terminal domain (residues 53–173) is shown on the left panel. For comparison, the root mean square fluctuation of the UIM-SH3 domains of STAM2 (PDB1X2Q) is shown on the right panel. The SH3 domain is a well characterized structural domain stable in time while the UIM domain is a highly flexible α-helix [77].
(EPS)

S3 Fig IglG is required for cytosolic localization of *F*. *novicida* at late time points post-infection. Vacuolar rupture and cytosolic localization were determined by the β-lactamase/CCF4 assay. J774 macrophages were infected for 18 h with the indicated *F*. *novicida* strains at an MOI of 100, incubated with CCF4 for 1 h and analyzed by flow cytometry. Upon release of *F*. *novicida* into the host cytosol, endogenous β-lactamase (FTN_1072)-mediated cleavage of the CCF4 probe leads to the appearance of Pacific-blue positive cells.
(EPS)

S4 Fig IglG is required for cytopathogenicity of *F*. *novicida* at late time points post-infection. Culture supernatants of infected J774 cells were assayed for LDH activity at the 0, 24 and 48 h time points and the activity was expressed as a percentage of the level of non-infected lysed cells (positive lysis control). Shown are means and SD of triplicate wells from one representative experiment of two. The asterisks indicate that the cytopathogenicity levels were different from those of U112-infected cells at a given time point as determined by a 2-sided *t*-test with equal variance (***, *P* ≤ 0.001).
(EPS)

S5 Fig Translocation of FPI proteins into macrophages using the β-lactamase/CCF4 assay. Left panel: J774 macrophages were infected with a Δ*bla* (Δ*FTN_1072*) mutant strain expressing the indicated TEM-fusion protein at an MOI of 100. At 18 h, macrophages were incubated with CCF4 for 1 h. TEM/β-lactamase-mediated CCF4 cleavage, indicative of the translocation of the fusion protein into the host cytosol, was analyzed by flow cytometry. Cells infected with the Δ*bla* mutant strain are presented as a negative control. PepO is a protein secreted in a T6SS-independent manner. Shown are means and SD of triplicate wells from one representative experiment of two. The asterisks indicate that the secretion levels were different from those of Δ*bla*-infected cells as determined by an unpaired *t*-test (two-tailed *P*-values are shown *, *P* ≤ 0.05; ***, *P* ≤ 0.001). Right panel: Western blot analysis using anti-TEM and anti-PdpB antibodies performed on lysates from a Δ*bla* mutant strain expressing the indicated TEM-fusion protein.
(EPS)

S6 Fig Conserved cysteines within IglG are required for IglG-mediated secretion of the Hcp homologue, IglC. The amount of IglC was assessed in the concentrated supernatant fractions or in bacterial pellets of the indicated strains grown in the presence or absence of 5% KCl. The non-secreted inner membrane protein PdpB was included as a control. One experiment representative of 2 independent experiments is shown.
(EPS)

S7 Fig The typical PAAR protein from *P*. *aeruginosa* (PA_0824) preferentially binds zinc. PA_0824 in fusion to GST was expressed in *E*. *coli* and purified by affinity chromatography followed by an anion exchange column. Metal content in the purified protein was analyzed by ICP-MS. Values from three independent purifications are shown.
(EPS)

S8 Fig The conformation of His_6_-IglG protein refolded in the presence of EDTA was analyzed by circular dichroism spectroscopy upon addition of increasing concentrations of MgSO_4_ and CoSO_4_ (concentrations are expressed as molar equivalent). (EPS)

S9 Fig Alignment of KU39_03731 from *Piscirickettsia salmonis* and IglG (FTN_1314) from *F*. *novicida*. Conserved cysteine residues are indicated (red arrows).
(EPS)

S10 Fig The IglG N-terminal extension protrudes from the PAAR-like domain. Structural superimposition of the IglG models generated by the I-Tasser (green, magenta, red, orange and wheat) and Phyre (cyan) homology servers. All models are represented as ribbons. The N-terminal predicted helices were modeled in various orientations by I-Tasser and not modelled by Phyre.
(EPS)

S11 Fig IglG is highly conserved between *Francisella* species. Alignment of IglG proteins from *F*. *novicida*, *F*. *tularensis* LVS and SCHU S4. Conserved cysteine residues are indicated (red arrows).
(EPS)

S12 Fig IglG expression in *F*. *tularensis* LVS. Analysis of IglG protein synthesis from LVS Δ*iglG* expressing 6 x His-tagged IglG or mutant forms thereof in *trans*. Proteins contained in the pellet fraction were separated by SDS-PAGE and identified by immunoblot using an anti-His antiserum. An antibody specific for IglB was used as a loading control. The experiment was repeated three times and a representative example is shown.
(EPS)

S13 Fig IglC-TEM1 and VgrG-TEM1 are secreted into macrophages by a Δ*iglG* LVS mutant although at much lower frequency than WT LVS. J774 macrophages were infected for 16h with the indicated strains, incubated with CCF4 and analyzed by flow cytometry. Concatenates from three samples are shown for each strain after exclusion of doublets and dead cells. Quantification is shown using the gating strategy presented on the FACS plots. Mean and SD of triplicates from one experiment are shown. Unpaired *t*-tests were performed; two-tailed *P*-values are shown (*, *P* ≤ 0.05; ***, *P* ≤ 0.001).
(EPS)

S14 Fig A Δ*FNI* mutant does not display any major loss of virulence *in vitro* or in a mouse model of tularemia. A: real time cell death of murine bone marrow derived macrophages infected at a MOI of 10 with the indicated strains. B: *In vivo* competitive indexes (CI) in a mouse model of tularemia. Mice were injected intradermally with 5 x 10^4^ cfu of two competitive strains (one kanamycin resistant and one sensitive). At 48h post-injection, spleens were collected and the bacterial counts were determined on TSA cysteine plates with and without kanamycin. CI was calculated as followed: CI = (Strain A/StrainB) in output/ (Strain A/Strain B) in input. To avoid any interference of the inherent kanamycin resistance with the assay, the CI displayed combines two independent CI: Δ*FNI*::Kan vs U112 and Δ*FNI* vs Δ*bla*::Kan. One sample *t*-tests were performed to analyze whether the experimental means were statistically different from 1 (ns: not significant, ***: *P* ≤0.001).
(PDF)

S1 Table Strains and plasmids used in this study. (DOCX)

S2 Table Oligonucleotides used in this study. (DOCX)

S3 Table Parameters used for ICP-MS-mediated metal concentration determination. (DOCX)

S1 Movie *In silico* simulation of IglG (residue 53–173) molecular dynamics with a 400ns trajectory. The time scale is shown by the coloration of IglG, which changes from red to blue over time.
(MPG)
