# Supplementary figures and images for: Francisella tularensis IglG Belongs to a Novel Family of PAAR-Like T6SS Proteins and Harbors a Unique N-terminal Extension Required for Virulence
Source: PLoS Pathog. 2016 Sep 7;12(9):e1005821. doi: 10.1371/journal.ppat.1005821 (PMC5014421; doi:10.1371/journal.ppat.1005821)

A

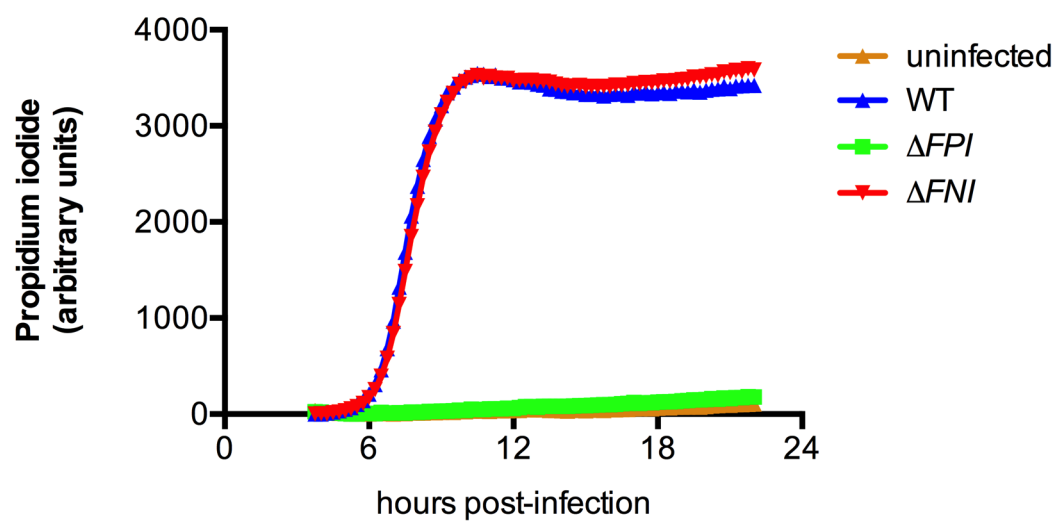

B

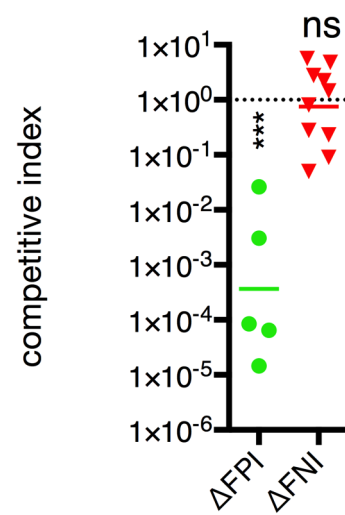

Figure S14

Supplement: S14 Fig — A: real time cell death of murine bone marrow derived macrophages infected at a MOI of 10 with the indicated strains. B: In vivo competitive indexes (CI) in a mouse model of tularemia. Mice were injected intradermally with 5 x 10^4^ cfu of two competitive strains (one kanamycin resistant and one sensitive). At 48h post-injection, spleens were collected and the bacterial counts were determined on TSA cysteine plates with and without kanamycin. CI was calculated as followed: CI = (Strain A/StrainB) in output/ (Strain A/Strain B) in input. To avoid any interference of the inherent kanamycin resistance with the assay, the CI displayed combines two independent CI: ΔFNI::Kan vs U112 and ΔFNI vs Δbla::Kan. One sample t-tests were performed to analyze whether the experimental means were statistically different from 1 (ns: not significant, ***: P ≤0.001). (PDF) [file ppat.1005821.s014.pdf]
