# Supplementary material for: Francisella tularensis IglG Belongs to a Novel Family of PAAR-Like T6SS Proteins and Harbors a Unique N-terminal Extension Required for Virulence
Source: PLoS Pathog. 2016 Sep 7;12(9):e1005821. doi: 10.1371/journal.ppat.1005821 (PMC5014421; doi:10.1371/journal.ppat.1005821)
Supplement: S1 Table — (DOCX) [file ppat.1005821.s015.docx]

**Table S1. Strains and plasmids used in this study**

| **Strain or plasmid** | **Relevant genotype or phenotype** | | **Source or reference** |
| --- | --- | --- | --- |
| **Strain** | |  |  |
| *E. coli* | |  |  |
| TOP10 | | F-*mcrA*, Δ(*mrr*-*hsdRMS-mcrBC*), φ80*lacZ*ΔM15, Δ*lacX*74, *recA1*, *deoR*, *araD*139, Δ(*ara-leu*)7679, *galU*, *galK*, *rpsL* (Str^R^), *endA1*, *nupG* | Invitrogen |
| S17-1λ*pir* | | *recA*, *thi*, *pro*, *hsdR^-^M^+^*, Sm^R^, <RP4:2-Tc:Mu:Ku:Tn7>Tp^R^ | [1] |
| DH5αF′IQ | | F-φ80*lac*ZΔM15 Δ(*lac*ZYA-*arg*F) U169 *rec*A1 *end*A1 *hsd*R17 (rk-, mk+) *pho*A *sup*E44 λ- *thi*-1 *gyr*A96 *rel*A1/F´ *pro*AB+ *lac*IqZΔM15 zzf::Tn5 [Km^R^]. | Invitrogen |
| KDZif1ΔZ | | B2H reporter strain, Km^R^, Cml^R^ | [2] |
| Rosetta | | F-*omp*T *hsd*S_B_(r_B_^-^ m_B_^-^) *gal dcm* λ(DE3 [*lac*I *lac*UV5-T7 gene 1 ind1 sam7 nin5]) pRARE (Cam^R^) | Novagen |
| *F. tularensis* subsp. *holarctica* | |  |  |
| LVS | | Live vaccine strain | USAMRIID^1^ |
| Δ*iglC* | | LVS, *iglC* in-frame deletion of codons 28-205 (the two copies have been deleted) | [3] |
| Δ*iglG* | | LVS, *iglG* in-frame deletion of codons 3-169 (the two copies have been deleted) | [4] |
| *F. tularensis* subsp. *tularensis* | |  |  |
| FSC237 | | SCHU S4, type A | FSC^2^ |
| Δ*iglC* | | FSC237, *iglC* in-frame deletion of codons 28-205 (the two copies have been deleted) | [5] |
| Δ*iglG* | | FSC237, *iglG* in-frame deletion of codons 3-169 (the two copies have been deleted) | This study |
| *F. tularensis* subsp. *novicida* | |  |  |
| U112  Δ*FPI*  Δ*FNI* | | Wild type  U112 Δ*FTN_1309-1325::aphA*  U112 Δ*FTN_0037-0054::FRTsc* | ATCC^3^  [6]  This study |
| Δ*iglG*  *Δbla*  *ΔvgrG* | | U112, *iglG* in-frame deletion of codons 3-169  U112, beta-lactamase in-frame deletion of codons 11-287  U112, Δ*FTN_1312::FRTsc, vgrG* in-frame deletion of codons 6-155 | This study  [7]  This study |
| **Plasmid** | |  |  |
| pCR^®^4-TOPO^®^  pUC19^®^  popHA  pGEX  pBS-II-KS+  pET151/D-TOPO^®^  pIglG-His  pGST-IglG | | TA cloning vector, Km^R^, Cb^R^  Cloning vector, Cb^R^  Cloning vector with HA tag, Cb^R^  pGEX-6-P3 cloning vector, Cb^R^  pBluescript-II-KS+ cloning vector, Cb^R^  TOPO cloning vector, Cb^R^  pET151/D-TOPO^®^, encoding IglG-6xHis, Cb^R^  pGEX-6-P3, encoding GST-IglG, Cb^R^ | Invitrogen  Invitrogen  P. Mangeot (unpublished) ^4^  GE Healthcare  Agilent technologies  Invitrogen  This study  This study |
| pGST-IglG_C64G_ | | pGEX-6-P3, encoding GST- IglG_C64G_, Cb^R^ | This study |
| pGST-IglG_C105G_ | | pGEX-6-P3, encoding GST- IglG_C105G_, Cb^R^ | This study |
| pGST-IglG_C122G_ | | pGEX-6-P3, encoding GST- IglG_C122G_, Cb^R^ | This study |
| pGST-IglG_C152G_ | | pGEX-6-P3, encoding GST- IglG_C152G_, Cb^R^ | This study |
| pGST-PA_0824 | | pGEX-6-P3, encoding GST- PA_0824, Cb^R^ | This study |
| pJEB753 | | pDM4 carrying a *Xho*I/*Sac*I PCR fragment of Δ*iglG*_3-169_ with flanking regions, Cm^R^ | [4] |
| pDMK2 | | Suicide plasmid carrying *sacBR*, Km^R^ | [8] |
| pJEB866 | | pDMK2 carrying a *Xho*I/*Sac*I PCR fragment of Δ*iglG*_3-169_ with flanking regions, Km^R^ | This study |
| pKK289Km | | expression plasmid carrying a *gfp* gene under the control of the LVS *groE* promoter, Km^R^ | [9] |
| pMOL103 | | pKK289Km, encoding IglG-6xHisC, Km^R^ | [4] |
| pJEB1040 | | pKK289Km, encoding IglG _C64A_-6xHisC, Km^R^ | This study |
| pJEB1041 | | pKK289Km, encoding IglG _C64S_-6xHisC, Km^R^ | This study |
| pJEB1042 | | pKK289Km, encoding IglG _C105A_-6xHisC, Km^R^ | This study |
| pJEB1043 | | pKK289Km, encoding IglG _C105S_-6xHisC, Km^R^ | This study |
| pJEB1044 | | pKK289Km, encoding IglG _C152A_-6xHisC, Km^R^ | This study |
| pJEB1045 | | pKK289Km, encoding IglG _C152S_-6xHisC, Km^R^ | This study |
| pJEB1060 | | pKK289Km, encoding IglG _C122A_-6xHisC, Km^R^ | This study |
| pJEB1061 | | pKK289Km, encoding IglG _C122S_-6xHisC, Km^R^ | This study |
| pJEB1062 | | pKK289Km, encoding IglG Δ_2-39_-6xHisC, Km^R^ | This study |
| pJEB733 | | pJEB709 encoding IglC-TEM1 | [10] |
| pMOL95 | | pJEB709 ecoding VgrG-TEM1 | [10] |
| pACTR-AP-Zif | | B2H vector, directs the synthesis of a Zif268-DNA binding domain fusion protein, Tet^R^ | [2] |
| pMOL135 | | pACTR-AP-Zif encoding IglA, Tet^R^ | [11] |
| pJEB828 | | pACTR-AP-Zif encoding IglF, Tet^R^ | [12] |
| pLEM27 | | pACTR-AP-Zif encoding IglG, Tet^R^ | [12] |
| pBRGPω | | B2H vector, directs the synthesis of a Gal11P-ω fusion protein, Cb^R^ | [2] |
| pMOL134 | | pBRGPω encoding IglB, Cb^R^ | [11] |
| pJEB829 | | pBRGPω encoding IglF, Cb^R^ | [12] |
| pLEM32 | | pBRGPω encoding IglG, Cb^R^ | [12] |
| pJEB1046 | | pBRGω encoding IglG _C64A_, Cb^R^ | This study |
| pJEB1047 | | pBRGω encoding IglG _C64S_, Cb^R^ | This study |
| pJEB1048 | | pBRGω encoding IglG _C105A_, Cb^R^ | This study |
| pJEB1049 | | pBRGω encoding IglG _C105S_, Cb^R^ | This study |
| pJEB1050 | | pBRGω encoding IglG _C152A_, Cb^R^ | This study |
| pJEB1051 | | pBRGω encoding IglG _C152S_, Cb^R^ | This study |
| pJEB1064 | | pBRGω encoding IglG _C122A_, Cb^R^ | This study |
| pJEB1065 | | pBRGω encoding IglG _C122S_, Cb^R^ | This study |
| pJEB1066 | | pBRGω encoding IglG Δ_2-39_, Cb^R^ | This study |
| pJEB1067 | | pBRGω encoding IglG Δ_58-173_, Cb^R^ | This study |
| pJEB1097 | | pBRGω encoding IglG Δ_68-173_, Cb^R^ | This study |
| pJEB1098 | | pBRGω encoding IglG Δ_78-173_, Cb^R^ | This study |
| pJEB1099 | | pBRGω encoding IglG Δ_88-173_, Cb^R^ | This study |
| pJEB1100  pJEB1123 | | pBRGω encoding IglG Δ_134-173_, Cb^R^  pBRGω encoding FTN_0054, Cb^R^ | This study  This study |
| pJEB1135 | | pBRGω encoding IglG _K10A_, Cb^R^ | This study |
| pJEB1136 | | pBRGω encoding IglG _R11A_, Cb^R^ | This study |
| pJEB1138 | | pBRGω encoding IglG _D19A_, Cb^R^ | This study |
| pJEB1139 | | pBRGω encoding IglG _E20A_, Cb^R^ | This study |
| pJEB1141 | | pBRGω encoding IglG _D28A_, Cb^R^ | This study |
| pJEB1142 | | pBRGω encoding IglG _D32A_, Cb^R^ | This study |
| pJEB1143 | | pBRGω encoding IglG-FTN_0054 hybrid, Cb^R^ | This study |
| pFNLTP6*gro-cyaA* | | pFNLTP6 expression plasmid carrying a *cyaA* gene under the control of the U112 *groE* promoter, Km^R^, Cb^R^ | [13][14] |
| pIglG-HA | | pFNLTP6 encoding IglG-HA, Km^R^, Cb^R^ | This study |
| pIglG_Δ2-17_ | | pFNLTP6 encoding IglG Δ_2-17_-HA, Km^R^, Cb^R^ | This study |
| pIglG_Δ2-39_ | | pFNLTP6 encoding IglG Δ_2-39_-HA, Km^R^, Cb^R^ | This study |
| pIglG_Δ2-58_ | | pFNLTP6 encoding IglG Δ_2-58_-HA, Km^R^, Cb^R^ | This study |
| pIglG_Δ2-66_ | | pFNLTP6 encoding IglG Δ_2-66_-HA, Km^R^, Cb^R^ | This study |
| pIglG_C64G_ | | pFNLTP6 encoding IglG_C64G_-HA, Km^R^, Cb^R^ | This study |
| pIglG_C105G_ | | pFNLTP6 encoding IglG_C105G_-HA, Km^R^, Cb^R^ | This study |
| pIglG_C122G_ | | pFNLTP6 encoding IglG_C122G_-HA, Km^R^, Cb^R^ | This study |
| pIglG_C152G_ | | pFNLTP6 encoding IglG_C152G_-HA, Km^R^, Cb^R^ | This study |
| pVgrG-HA | | pFNLTP6 encoding VgrG-HA, Km^R^, Cb^R^ | This study |
| pFNLTP6-TEM | | pFNLTP6 encoding TEM, Km^R^ | This study |
| pIglG-TEM | | pFNLTP6 encoding IglG-TEM, Km^R^ | This study |
| pVgrG-TEM | | pFNLTP6 encoding VgrG-TEM, Km^R^ | This study |
| pIglC-TEM | | pFNLTP6 encoding IglC-TEM, Km^R^ | This study |
| pIglI-TEM | | pFNLTP6 encoding IglI-TEM, Km^R^ | This study |
| pPepO-TEM | | pFNLTP6 encoding PepO-TEM, Km^R^ | This study |
| pPdpE-TEM | | pFNLTP6 encoding PdpE-TEM, Km^R^ | This study |
| pIglF-TEM | | pFNLTP6 encoding IglF-TEM, Km^R^ | This study |

^1^ US Army Medical Research Institute of Infectious Diseases, Fort Detrick, Frederick, MD

^2^ FSC, Francisella Strain Collection, Swedish Defence Research Agency, Umeå

^3^ ATCC, American Type Culture Collection, Manassas, VA, USA

^4^ Plasmid sequence can be obtained upon request to Philippe Mangeot (Centre International de Recherche en Infectiology, Lyon, France).

References:

1. Simon R, Priefer U, Puhler A. A Broad Host Range Mobilization System for In Vivo Genetic Engineering: Transposon Mutagenesis in Gram Negative Bacteria. Nat Biotech. 1983;1: 784–791. doi:10.1038/nbt1183-784

2. Vallet-Gely I, Donovan KE, Fang R, Joung JK, Dove SL. Repression of phase-variable cup gene expression by H-NS-like proteins in Pseudomonas aeruginosa. Proc Natl Acad Sci U S A. 2005;102: 11082–11087. doi:10.1073/pnas.0502663102

3. Golovliov I, Baranov V, Krocova Z, Kovarova H, Sjostedt A. An attenuated strain of the facultative intracellular bacterium Francisella tularensis can escape the phagosome of monocytic cells. Infect Immun. 2003;71: 5940–50.

4. Broms JE, Lavander M, Meyer L, Sjostedt A. IglG and IglI of the Francisella pathogenicity island are important virulence determinants of Francisella tularensis LVS. Infect Immun. 2011;79: 3683–3696. doi:10.1128/IAI.01344-10

5. Twine S, Bystrom M, Chen W, Forsman M, Golovliov I, Johansson A, et al. A mutant of Francisella tularensis strain SCHU S4 lacking the ability to express a 58-kilodalton protein is attenuated for virulence and is an effective live vaccine. Infect Immun. 2005;73: 8345–8352. doi:10.1128/IAI.73.12.8345-8352.2005

6. Weiss DS, Brotcke A, Henry T, Margolis JJ, Chan K, Monack DM. In vivo negative selection screen identifies genes required for Francisella virulence. Proc Natl Acad Sci U A. 2007;104: 6037–42.

7. Meunier E, Wallet P, Dreier RF, Costanzo S, Anton L, Ruhl S, et al. Guanylate-binding proteins promote activation of the AIM2 inflammasome during infection with Francisella novicida. Nat Immunol. 2015;16: 476–484. doi:10.1038/ni.3119

8. Kadzhaev K, Zingmark C, Golovliov I, Bolanowski M, Shen H, Conlan W, et al. Identification of genes contributing to the virulence of Francisella tularensis SCHU S4 in a mouse intradermal infection model. PloS One. 2009;4: e5463. doi:10.1371/journal.pone.0005463

9. Bonquist L, Lindgren H, Golovliov I, Guina T, Sjostedt A. MglA and Igl proteins contribute to the modulation of Francisella tularensis live vaccine strain-containing phagosomes in murine macrophages. Infect Immun. 2008;76: 3502–3510. doi:10.1128/IAI.00226-08

10. Broms JE, Meyer L, Sun K, Lavander M, Sjostedt A. Unique substrates secreted by the type VI secretion system of Francisella tularensis during intramacrophage infection. PLoS One. 2012;7: e50473. doi:10.1371/journal.pone.0050473

11. Broms JE, Meyer L, Lavander M, Larsson P, Sjostedt A. DotU and VgrG, core components of type VI secretion systems, are essential for Francisella LVS pathogenicity. PloS One. 2012;7: e34639. doi:10.1371/journal.pone.0034639

12. Lindgren M, Broms JE, Meyer L, Golovliov I, Sjostedt A. The Francisella tularensis LVS DeltapdpC mutant exhibits a unique phenotype during intracellular infection. BMC Microbiol. 2013;13: 20. doi:10.1186/1471-2180-13-20

13. Maier TM, Havig A, Casey M, Nano FE, Frank DW, Zahrt TC. Construction and characterization of a highly efficient Francisella shuttle plasmid. Appl Environ Microbiol. 2004;70: 7511–7519. doi:10.1128/AEM.70.12.7511-7519.2004

14. Clemens DL, Lee BY, Horwitz MA. Francisella tularensis phagosomal escape does not require acidification of the phagosome. Infect Immun. 2009;77: 1757–73. doi:10.1128/IAI.01485-08
