# Supplementary material for: Francisella tularensis IglG Belongs to a Novel Family of PAAR-Like T6SS Proteins and Harbors a Unique N-terminal Extension Required for Virulence
Source: PLoS Pathog. 2016 Sep 7;12(9):e1005821. doi: 10.1371/journal.ppat.1005821 (PMC5014421; doi:10.1371/journal.ppat.1005821)
Supplement: S2 Table — (DOCX) [file ppat.1005821.s016.docx]

**TABLE S2. Oligonucleotides used in this study**

| Purpose | Oligonucleotide pair(s) |
| --- | --- |
| *U112 deletion mutant*  *ΔFNI*: Δ*FTN_0037-0054*  IglG Δ_2-162_  VgrG Δ_6-155_  *Complementation* | (1) *FNI* upstream region: Up_FNI_For: 5’- TGG GAT AAC CAC TAA AAT CCA TGC-3’ + Up_FNI_FRT_rev : 5’-G CTT ATC GAT ACC GTC GAC CTC CTC TAA GCT TAT CTC TAA CAA GCA TTT TAG TAG-3’  (2) *FNI* downstream region : FRT_Down_FNI_For : 5’- GAT ATC GAT CCT GCA GCT ATG CGG TAA GGT TAT TAC GAT TGC ACT AGC-3’ + Down_FNI_Rev :5’- GGA CCC ACT ATC AGC TCA CCT G-3’  (3) Kan_FRT_cassette_For : 5’-GAG GTC GAC GGT ATC GAT AAG C-3’ + Kan_FRT_cassette_Rev :5’-  GCA TAG CTG CAG GAT CGA TAT C-3’  (1) *iglG* upstream region: Up_IglG_For: 5’- C TTC CAA ATA CTA CTA GAC ATA GCA ACA ATG-3’ + Up_IglG_FRT_rev : 5’-G CTT ATC GAT ACC GTC GAC CTC AAC ATT TAA ATT TTC CAA TAA GCT TCT TGC-3’  (2) *iglG* downstream region : FRT_Down_IglG_For : 5’-GAT ATC GAT CCT GCA GCT ATG CGT GGT GGA CAA ATA AAT GTA AAA ACA TCT-3’ + Down_IglG_Rev :5’-T CAA AGT TAT TTG CAA ATT CCA ATG-3’  (3) Kan_FRT_cassette_For  + Kan_FRT_cassette_Rev  1) *vgrG* upstream region: Up_VgrG_For: 5’- GTA CAA CTG GAA TTC ACT TG-3’ + Up_VgrG_FRT_rev : 5’-G CTT ATC GAT ACC GTC GAC CTC GTC TGC TTT TGA CAT AAG AAT-3’  (2) *vgrG* downstream region : FRT_Down_VgrG_For : 5’-GAT ATC GAT CCT GCA GCT ATG CGG TTC CGC AGC AAC AAT GGT-3’ + Down_VgrG_Rev :5’-ATA TCG CTA GCT AAA AGA CG-3’  (3) Kan_FRT_cassette_For + Kan_FRT_cassette_Rev |
| IglG _C64A_-6xHis | IglG_NdeI_F: 5´-*CAT ATG* TTA AAT ATT ATA AAT GAC TCC T (*Nde*I) + IglG_C64A_b: 5´-**TGC** TTT AAT TTG TGC TCC CAT TGA TG-3´  IglG_C64A_c: 5´-A GCA CAA ATT AAA **GCA** TCA TAT AGT CTT GGA TCA GGA-3´ + pigD_6xH_rev_C: 5´-*GAA TTC* CTA ATG ATG ATG ATG ATG ATG AGA TGT TTT TAC ATT TAT TTG TC-3´ (*Eco*RI) |
| IglG _C64S_-6xHis | IglG_NdeI_F (*Nde*I) + IglG_C64S_b: 5´- **TGA** TTT AAT TTG TGC TCC CAT TGA TG-3´  IglG_C64S_c: 5´-A GCA CAA ATT AAA **TCA** TCA TAT AGT CTT GGA TCA GGA-3´ + pigD_6xH_rev_C (*Eco*RI) |
| IglG _C105A_-6xHis | IglG_NdeI_F (*Nde*I) + IglG_C105A_b: 5´-**TGC** TCC AGC AAA AGG TAA AAT GTT AG-3´  IglG_C105A_c: 5´-A CCT TTT GCT GGA **GCA** ACA AAT CCA GCA AAC CCT AC-3´ + pigD_6xH_rev_C (*Eco*RI) |
| IglG _C105S_-6xHis | IglG_NdeI_F (*Nde*I) + IglG_C105S_b: 5´-**TGA** TCC AGC AAA AGG TAA AAT GTT AG-3´  IglG_C105S_c: 5´-A CCT TTT GCT GGA **TCA** ACA AAT CCA GCA AAC CCT AC-3´ + pigD_6xH_rev_C (*Eco*RI) |
| IglG _C122A_-6xHis | IglG_NdeI_F (*Nde*I) + IglG_C122A_b:5´-**TGC** TAC CCA AGG AAA GCT AAA TGG-3´  IglG_C122A_c: 5´-C TTT CCT TGG GTA **GCA** ATA CCA AAC TTA TCT GCT TTT A-3´ + pigD_6xH_rev_C (*Eco*RI) |
| IglG _C122S_-6xHis | IglG_NdeI_F (*Nde*I) + IglG_C122S_b: 5´-**TGA** TAC CCA AGG AAA GCT AAA TGG-3´  IglG_C122S_c: 5´-C TTT CCT TGG GTA **TCA** ATA CCA AAC TTA TCT GCT TTT A-3´ + pigD_6xH_rev_C (*Eco*RI) |
| IglG _C152A_-6xHis | IglG_NdeI_F (*Nde*I) + IglG_C152A_b: 5´-**TGC** CAT TGC TTT ACT ATT TAT TGT AG-3´  IglG_C152A_c: 5´-T AGT AAA GCA ATG **GCA** ATG TTT GCT CCG GGT GGT A-3´ + pigD_6xH_rev_C (*Eco*RI) |
| IglG _C152S_-6xHis | IglG_NdeI_F (*Nde*I) + IglG_C152S_b: 5´-**TGA** CAT TGC TTT ACT ATT TAT TGT AG-3´  IglG_C152S_c: 5´-T AGT AAA GCA ATG **TCA** ATG TTT GCT CCG GGT GGT A-3´ + pigD_6xH_rev_C (*Eco*RI) |
| IglG Δ_2-39_-6xHis | IglG_DN_F*:* 5´*-CAT ATG* TTA GCT CAA TCA AAA CTA CAC TT-3´ (*Nde*I) + pigD_6xH_rev_C (*Eco*RI) |
| IglG-HA in popHA | IglG_XhoI_F: 5´-G CGC *CTC GAG* ATG TTA AAT ATT ATA AAT GAC TCC T-3´ (*Xho*I) + IglG_Hind3_rev: 5´-G CGC *AAG CTT* AGA TGT TTT TAC ATT TAT TTG TCC-3´ (*Hind*III); |
| IglG-HA in pFNLTP6 | SD_IglG_a_F: 5´-A AGG AGT AAT TTC ATG TTA AAT ATT ATA AAT GAC TCC T-3´ + 2HAmod_BamH1_Rev: 5´-G CGC *GGA TCC* TCA CTG CAG GGA TGC GTA GTC AGG CAC GTC GTA TGG ATA AGA G-3´ (*Bam*HI); SD_ATG_Nde1_F: 5´-TT T*CA TAT G*CC TAT TTA AGG AGT AAT TTC ATG-3´ (*Nde*I) + 2HAmod_BamH1_Rev (*Bam*HI) |
| IglG Δ_2-17_-HA | Δ2-17_IglG_F: 5´-CC TAT TTA AGG AGT AAT TTC ATG GAT GAT GAA AGT ATT AGT AGC TCA GTA TCT G-3´ + 2HAmod_BamH1_Rev (*Bam*HI); + SD_ATG_Nde1_F (*Nde*I) + 2HAmod_BamH1_Rev (*Bam*HI) |
| IglG Δ_2-39_-HA | Δ2-39_IglG_F: 5´-CC TAT TTA AGG AGT AAT TTC ATG TTA GCT CAA TCA AAA CTA CAC TTA TCA AGC-3´+ 2HAmod_BamH1_Rev (*Bam*HI); + SD_ATG_Nde1_F (*Nde*I) + 2HAmod_BamH1_Rev *(Bam*HI) |
| IglG Δ_2-58_-HA | Δ2-58_IglG_F: 5´-CC TAT TTA AGG AGT AAT TTC ATG GGA GCA CAA ATT AAA TGC TCA TAT AGT CTT G-3´+ 2HAmod_BamH1_Rev (BamHI); + SD_ATG_Nde1_F (*Nde*I) + 2HAmod_BamH1_Rev (*Bam*HI) |
| IglG Δ_2-66_-HA | Δ2-66_IglG_F: 5´-CC TAT TTA AGG AGT AAT TTC ATG AGT CTT GGA TCA GGA ATT TAT CTT AGT AC -3´ + 2HAmod_BamH1_Rev (*Bam*HI); + SD_ATG_Nde1_F (*Nde*I) + 2HAmod_BamH1_Rev (*Bam*HI) |
| VgrG-HA | SD_VgrG_F: 5’-A AGG AGT AAT TTC ATG TCA AAA GCA GAC CAT ATT TTC AAC TTA GAA G-3’ + VgrG_Nhe1_Rev 5’-TTT *GCT AGC* TCC AAC CAT TGT TGC TGC GGA ACC-3’ *(Nhe1) ;*  SD-ATG-EcoRI_F: 5´-A TAT T*GA ATT C*CC TAT TTA AGG AGT AAT TTC ATG-3´ (*Eco*RI) + VgrG_Nhe1_Rev *(Nhe1)* |
| IglG in pBS-II-KS+ | SD_IglG_a_F + 2HAmod_BamH1_Rev (*Bam*HI); SD-ATG-EcoRI_F: 5´-A TAT T*GA ATT C*CC TAT TTA AGG AGT AAT TTC ATG-3´ (*Eco*RI) + 2HAmod_BamH1_Rev (*Bam*HI) |
| IglG_C64G_ | IglG_C64G_F: 5´-CA ACG GGA GCA CAA ATT AAA **GGC** TCA TAT AGT CTT GGA TCA GG-3´ + IglG_C64G_Rev: 5´-CC TGA TCC AAG ACT ATA TGA **GCC** TTT AAT TTG TGC TCC CGT TG-3´ |
| IglG_C105G_ | IglG_C105G_F: 5´-GCT AAC ATT TTA CCT TTT GCT GGA **GGT** ACA AAT CCA GCA AAC CC-3´ + IglG_C105G_Rev: 5´-GG GTT TGC TGG ATT TGT **ACC** TCC AGC AAA AGG TAA AAT GTT AGC-3´ |
| IglG_C122G_ | IglG_C122G_F: 5´-CCA TTT AGC TTT CCT TGG GTA **GGT** ATA CCA AAC TTA TCT GC-3´ + IglG_C122G_Rev: 5´-GC AGA TAA GTT TGG TAT **ACC** TAC CCA AGG AAA GCT AAA TGG-3´ |
| IglG_C152G_ | IglG_C152G_F: 5´-CT ACA ATA AAT AGT AAA GCA ATG **GGT** ATG TTT GCT CCG GGT GG-3´ + IglG_C152G_Rev: 5´-CC ACC CGG AGC AAA CAT **ACC** CAT TGC TTT ACT ATT TAT TGT AG-3´ |
| *TEM - translocation* |  |
| Deletion of Beta-lactamase in pFNLTP6 | KanR_pstI_F: 5´-AA *CTG CAG* GAC GAG GCA GCG-3´ (*Pst*I) + KanR_Tail_ColE1_Rev: 5´-GGA ACG AAA ACT CAC GTT AAG GGA TTT TGG TCA TGA TCA GAA GAA CTC GTC AAG AAG GCG ATA G-3´; ColE1_F: 5´-TCA TGA CCA AAA TCC CTT AAC GTG AGT TTT CG-3´+ Gro_promotor_NdeI_Rev: 5´-ATT *CAT ATG* GTA CCT GCA CGA CGA ACT AAT AC-3´ (*Nde*I) |
| E.coli TEM in pFNLTP6 | E.coli_TEM_F: 5´-*GCT AGC* ACG CGT GTT AAC CAC CCA GAA ACG CTG GTG AAA GTA AAA G-3´ (*Nhe*I) + E.coli_TEM_Rev: 5´-TTT *GGA TCC* TTA CCA ATG CTT AAT CAG TGA GGC ACC TAT C-3´ (*Bam*HI) |
| IglG-TEM | SD_IglG_b_F: 5´-A AGG AGT AAT TTC ATG TTA AAT ATT ATA AAT GAC TCC TTA AAA CGC TTG G-3´ + IglG_Nhe1_Rev: 5´-TTT *GCT AGC* AGA TGT TTT TAC ATT TAT TTG TCC ACC ACT AAT AAA ATC-3´ (*Nhe*I); SD-ATG-EcoRI_F *(Eco*RI) + IglG_Nhe1_Rev (*Nhe*I) |
| VgrG-TEM | SD_VgrG_EcoRI_F: 5´-AA TTT *GAA TTC* GCA GCA TAG AAA AAG ATT AAG GGG ATA TTC-3´ (*Eco*RI) + VgrG_NheI_Rev: 5´-TTT *GCT AGC* TCC AAC CAT TGT TGC TGC GGA ACC-3´ (*Nhe*I) |
| IglC-TEM | SD_IglC_EcoRI_F: 5´-CG TTG *GAA TTC* GAT ACT AGG CTT GAA CCA GAA TTA TTC GGT AC-3´ (*Eco*RI) + IglC_NotI_Rev: 5´-TT T*GC GGC CGC* TGC AGC TGC AAT ATA TCC TAT TTT AGC AAC-3´ (*Not*I) |
| IglI-TEM | SD_IglI_EcoRI_F: 5´-AA TTT *GAA TTC CGT TCC TTT AGG ACTG TTT CTT TTA AGC TTA C*-3´(*Eco*RI) + IglI_NotI_Rev: 5´-TT T*GC GGC CGC* TAT GTC AAA AAG ATC TTC AAA ATA GTC TTC ATC TAT CT-3´ (*Not*I) |
| PepO-TEM | SD_PepO_EcoRI_F: 5´-CG TTG *GAA TTC* CTT AGG ATT ATT AAC TTT GTT GGC ATC ATA TGT AGC-3´ (*Eco*RI) + PepO_NotI_Rev: 5´-TT T*GC GGC CGC* CCA GAT ATT AAC TCT TTG TTC TGG ATC TAA GTA CA-3´ (*Not*I) |
| PdpE-TEM | SD_PdpE_EcoRI_F: 5´-AA TTT *GAA TTC* GAA ATC AAT CAT AAC TAA TAC AAC CTC GTT ACT TTA TGA C-3´ (*Eco*RI) + PdpE_NheI_Rev: 5´-TTT *GCT AGC* TAT TAT AGT AAT TTT CTT TTC ATA ATG AGG GAT AGC CCA-3´ (*Nhe*I) |
| IglF-TEM | SD_IglF_HA_F: 5´-A AGG AGT AAT TTC **ATG** AAT AAT GAT ATT GAT AAA TGG TTT GAA AAT TTA TTT AAC AAT ATC C-3´ + IglF_NotI_Rev: 5´-ATA T*GC GGC CGC* AAT TTT CCA ATA AGC TTC TTG CTT GCT TTT TAT AC-3´ (*Not*I); SD_ATG_Nde1_F (*Nde*I) + IglF_NotI_Rev (*Not*I) |
| *E.coli expression* |  |
| GST-IglG (and cysteine mutants) | IglG_BamHI_F: 5´-TTT *GGA TCC* ATG TTA AAT ATT ATA AAT GAC TCC TTA AAA CGC-3´ (*Bam*HI) + IglG_EcoRI_ Rev: 5´- CG TTG *GAA TTC* CTA AGA TGT TTT TAC ATT TAT TTG TCC ACC-3´ (*Eco*RI) |
| GST_PA_0824 | PA_0824_BamHI_F: 5'-TTT*GGATCC* ATGAGTGGAAAACCCGCCGCCC-3' *(BamHI)* +  PA_0824_EcoRI_R: 5'- CGTTG*GAATTC*TCATGCGTCGCACCTCTGCACC-3' *(EcoRI)* |
| His_6_-IglG | IglG_NcoI_F: 5' -CA*CCATGG*GATTAAATATTATAAATGACTCCTTAAAACGCTTGG-3' (*NcoI)* +  IglG_BamHI_R: 5'- gcgc*ggatcc*ctaagatgtttttacatttatttgtccaccac-3' (*BamHI)* |
| *B2H expression* |  |
| IglG _C64A_ | IglG_NdeI_F (*Nde*I) + IglG_C64A_b  IglG_C64A_c + IglG_NotI_R: 5´-*GCG GCC GC* AGA TGT TTT TAC ATT TAT TTG TCC A-3´ (*Not*I) |
| IglG _C64S_ | IglG_NdeI_F (*Nde*I) + IglG_C64S_b  IglG_C64S_c + IglG_NotI_R (*Not*I) |
| IglG _C105A_ | IglG_NdeI_F (*Nde*I) + IglG_C105A_b  IglG_C105A_c + IglG_NotI_R (*Not*I) |
| IglG _C105S_ | IglG_NdeI_F (*Nde*I) + IglG_C105S_b  IglG_C105S_c + IglG_NotI_R (*Not*I) |
| IglG _C122A_ | IglG_NdeI_F (*Nde*I) + IglG_C122A_b  IglG_C122A_c + IglG_NotI_R (*Not*I) |
| IglG _C122S_ | IglG_NdeI_F (*Nde*I) + IglG_C122S_b  IglG_C122S_c + IglG_NotI_R (*Not*I) |
| IglG _C152A_ | IglG_NdeI_F (*Nde*I) + IglG_C152A_b  IglG_C152A_c + IglG_NotI_R (*Not*I) |
| IglG _C152S_ | IglG_NdeI_F (*Nde*I) + IglG_C152S_b  IglG_C152S_c + IglG_NotI_R (*Not*I) |
| IglG Δ_2-39_ | IglG_DN_F (*Nde*I) + IglG_NotI_R (*Not*I) |
| IglG Δ_58-173_ | IglG_NdeI_F (*Nde*I) + IglG_DC_NotIR: 5´-*GC GGC CGC* TGA TGT GGT TAA TAT TGA CGC A-3´ (*Not*I) |
| IglG Δ_68-173_ | IglG_NdeI_F (*Nde*I) + D68-173_NotIR: 5´-*GC GGC CGC* ACT ATA TGA GCA TTT AAT TTG TGC-3´ (*Not*I) |
| IglG Δ_78-173_ | IglG_NdeI_F (*Nde*I) + D78-173_NotIR: 5´-*GC GGC CGC* TCT TGT ACT AAG ATA AAT TCC TG-3´ (*Not*I) |
| IglG Δ_88-173_ | IglG_NdeI_F (*Nde*I) + D88-173_NotIR: 5´-*GC GGC CGC* AGC TGG TAA GTT GTT CGT TAG-3´ (*Not*I) |
| IglG Δ_134-173_ | IglG_NdeI_F (*Nde*I) + D134-173_NotIR: 5´-*GC GGC CGC* GTT CGT GGG GAT AAA AGC AG-3´ (*Not*I) |
| IglG _K10A_ | IglG_K_F: 5´-*CAT ATG* TTA AAT ATT ATA AAT GAC TCC TTA **GC**A CGC TTG AAA G-3´ (*Nde*I) + IglG_NotI_R (*Not*I) |
| IglG _R11A_ | IglG_R_F: 5´-*CAT ATG* TTA AAT ATT ATA AAT GAC TCC TTA AAA **GCA** TTG AAA GAA ATA AC-3´ (*Nde*I) + IglG_NotI_R (*Not*I) |
| IglG _D19A_ | IglG_NdeI_F (*Nde*I) + IglG_D_b: 5´-A**G**C ATT GGT CGT TAT TTC TTT CAA GC-3´ IglG_D_c: 5´-ATA ACG ACC AAT G**C**T GAA AGT ATT AGT AGC TCA G-3´ + IglG_NotI_R (*Not*I) |
| IglG _E20A_ | IglG_NdeI_F (*Nde*I) + IglG_E_b: 5´-T**G**C ATC ATT GGT CGT TAT TTC TTT CA-3´  IglG_E_c: 5´-A ACG ACC AAT GAT G**C**A AGT ATT AGT AGC TCA GTA-3´ + IglG_NotI_R (*Not*I) |
| IglG _D28A_ | IglG_NdeI_F (*Nde*I) + IglG_D28_b: 5´-A**G**C AGA TAC TGA GCT ACT AAT ACT TTC-3´ IglG_D28_c: 5´-T AGC TCA GTA TCT G**C**T CTA GTG GCG GAT TTA AAC-3´ + IglG_NotI_R (*Not*I) |
| IglG _D32A_ | IglG_NdeI_F (*Nde*I) +IglG_D32_b: 5´-A**G**C CGC CAC TAG ATC AGA TAC TG-3´ IglG_D32_c: 5´-GAT CTA GTG GCG G**C**T TTA AAC AAT ATA AAA ATT TTA TTA G-3´ + IglG_NotI_R (*Not*I) |
| IglG-FTN_0054 hybrid | IglG_NdeI_F (*Nde*I) + Hybrid_b: 5´-AA AGA ACA TCT TAT CAA TGC TCC CAT TGA TGT GGT TAA TAT TGA CG-3´ Hybrid_c: 5´-TA ACC ACA TCA ATG GGA GCA TTG ATA AGA TGT TCT TTT GGA GCT AC-3´ + FTN_0054_R (*Not*I) |
|  |  |

The nucleotide sequences in italics represent the incorporated *Nde*I, *Eco*RI, *Not*I, *Bam*HI, *Nco*I, and *Nhe*I restriction sites used for cloning of the PCR amplified DNA fragments. The underlined sequence indicates the complementary overlap between respective primers in the overlap PCR reactions. In primers used to generate amino acid substitutions, the nucleotides substituted are indicated in boldface. To optimize expression, all substitutions were adapted according to the codon usage preferences of *F*. *tularensis* (http://www.kazusa.or.jp/codon).
