# Supplementary material for: Francisella tularensis IglG Belongs to a Novel Family of PAAR-Like T6SS Proteins and Harbors a Unique N-terminal Extension Required for Virulence
Source: PLoS Pathog. 2016 Sep 7;12(9):e1005821. doi: 10.1371/journal.ppat.1005821 (PMC5014421; doi:10.1371/journal.ppat.1005821)
Supplement: S3 Table — (DOCX) [file ppat.1005821.s017.docx]

**Table S3. Parameters used for ICP-MS-mediated metal concentration determination**

RF power (W) 1350

Ar Flow Rates (l/min) Cool 13.5, Aux 0.8 and Nebulizer 1.0

Sample uptake rate 200 µl/min

Isotopes ^64^Zn, ^66^Zn, ^67^Zn, ^56^Fe, ^57^Fe, ^115^In

Internal Standard ^115^In

Acquisition mode Helium in collision cell

Tuning sensitivity 450 000 cps/ppb In in standard mode
